# TRMT10A dysfunction perturbs codon translation of initiator methionine and glutamine and impairs brain functions in mice

**DOI:** 10.1093/nar/gkae520

**Published:** 2024-07-02

**Authors:** Roland Tresky, Yuta Miyamoto, Yu Nagayoshi, Yasushi Yabuki, Kimi Araki, Yukie Takahashi, Yoshihiro Komohara, Huicong Ge, Kayo Nishiguchi, Takaichi Fukuda, Hitomi Kaneko, Nobuko Maeda, Jin Matsuura, Shintaro Iwasaki, Kourin Sakakida, Norifumi Shioda, Fan-Yan Wei, Kazuhito Tomizawa, Takeshi Chujo

**Affiliations:** Department of Molecular Physiology, Faculty of Life Sciences, Kumamoto University, Kumamoto 860-8556, Japan; Department of Anatomy and Neurobiology, Faculty of Life Sciences, Kumamoto University, Kumamoto 860-8556, Japan; Department of Molecular Physiology, Faculty of Life Sciences, Kumamoto University, Kumamoto 860-8556, Japan; Department of Nephrology, Faculty of Life Sciences, Kumamoto University, Kumamoto 860-8556, Japan; Department of Genomic Neurology, Institute of Molecular Embryology and Genetics, Kumamoto University, Kumamoto 860-0811, Japan; Division of Developmental Genetics, Institute of Resource Development and Analysis, Kumamoto University, Kumamoto 860-0811, Japan; Department of Anatomy and Neurobiology, Faculty of Life Sciences, Kumamoto University, Kumamoto 860-8556, Japan; Department of Cell Pathology, Faculty of Life Sciences, Kumamoto University, Kumamoto 860-8556, Japan; Department of Molecular Physiology, Faculty of Life Sciences, Kumamoto University, Kumamoto 860-8556, Japan; Department of Molecular Physiology, Faculty of Life Sciences, Kumamoto University, Kumamoto 860-8556, Japan; Department of Nephrology, Faculty of Life Sciences, Kumamoto University, Kumamoto 860-8556, Japan; Department of Anatomy and Neurobiology, Faculty of Life Sciences, Kumamoto University, Kumamoto 860-8556, Japan; Department of Molecular Physiology, Faculty of Life Sciences, Kumamoto University, Kumamoto 860-8556, Japan; Department of Gastroenterology and Hepatology, Faculty of Life Sciences, Kumamoto University, Kumamoto 860-8556, Japan; Department of Molecular Physiology, Faculty of Life Sciences, Kumamoto University, Kumamoto 860-8556, Japan; Department of Neurosurgery, Faculty of Life Sciences, Kumamoto University, Kumamoto 860-8556, Japan; RNA Systems Biochemistry Laboratory, RIKEN Cluster for Pioneering Research, Saitama 351-0198, Japan; Department of Computational Biology and Medical Sciences, Graduate School of Frontier Sciences, The University of Tokyo, Chiba 277-8561, Japan; Department of Molecular Physiology, Faculty of Life Sciences, Kumamoto University, Kumamoto 860-8556, Japan; Department of Metabolic Medicine, Faculty of Life Sciences, Kumamoto University, Kumamoto 860-8556, Japan; Department of Genomic Neurology, Institute of Molecular Embryology and Genetics, Kumamoto University, Kumamoto 860-0811, Japan; Department of Modomics Biology and Medicine, Institute of Development, Aging and Cancer, Tohoku University, Sendai 980-8575, Japan; Department of Molecular Physiology, Faculty of Life Sciences, Kumamoto University, Kumamoto 860-8556, Japan; Department of Molecular Physiology, Faculty of Life Sciences, Kumamoto University, Kumamoto 860-8556, Japan

## Abstract

In higher eukaryotes, tRNA methyltransferase 10A (TRMT10A) is responsible for *N^1^*-methylguanosine modification at position nine of various cytoplasmic tRNAs. Pathogenic mutations in *TRMT10A* cause intellectual disability, microcephaly, diabetes, and short stature in humans, and generate cytotoxic tRNA fragments in cultured cells; however, it is not clear how TRMT10A supports codon translation or brain functions. Here, we generated *Trmt10a* null mice and showed that tRNA^Gln(CUG)^ and initiator methionine tRNA levels were universally decreased in various tissues; the same was true in a human cell line lacking TRMT10A. Ribosome profiling of mouse brain revealed that dysfunction of TRMT10A causes ribosome slowdown at the Gln(CAG) codon and increases translation of *Atf4* due to higher frequency of leaky scanning of its upstream open reading frames. Broadly speaking, translation of a subset of mRNAs, especially those for neuronal structures, is perturbed in the mutant brain. Despite not showing discernable defects in the pancreas, liver, or kidney, *Trmt10a* null mice showed lower body weight and smaller hippocampal postsynaptic densities, which is associated with defective synaptic plasticity and memory. Taken together, our study provides mechanistic insight into the roles of TRMT10A in the brain, and exemplifies the importance of universal tRNA modification during translation of specific codons.

## Graphical Abstract

**Graphical Abstract:**
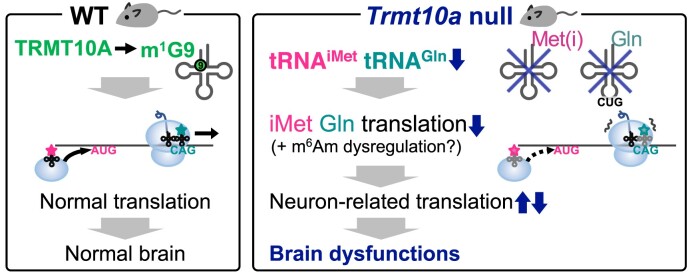

## Introduction

tRNA is an adaptor molecule that translates genetic information transcribed on mRNA to generate proteins (1,2). tRNAs are post-transcriptionally decorated with a variety of chemical modifications that are incorporated by specific modifying enzymes (3). These tRNA modifications are important for maintaining tRNA structural integrity, biochemical stability, and/or appropriate codon–anticodon interactions (4). In humans, tRNAs collectively contain >40 different modifications at specific positions, and the importance of the modifications is emphasized by the existence of over 50 human tRNA modification enzymes harboring pathogenic mutations (5). Such ‘RNA modopathies’ or ‘tRNA modopathies’ frequently manifest as brain dysfunction, cancer, diabetes, or mitochondrial diseases (4–7).

Among the phenotypes of human tRNA modopathies, brain dysfunction is associated with the largest number (>20) of tRNA modification enzymes harboring pathogenic mutations. However, the reason why the brain is particularly susceptible to the loss of tRNA modifications is poorly understood due to the necessity to generate animal models. A recent study revealed that whole-body knockout of *Ftsj1* (a gene encoding a 2′-*O*-methyltransferase for tRNA positions 32 and 34) in mice caused degradation of tRNA^Phe^ only among the various tRNAs methylated by FTSJ1, thereby slowing down Phe codon translation in the brain (8). Intriguingly, tRNA^Phe^ degradation occurred only in the brain and not in other mouse tissues, such as the kidney and liver, which partly explains the brain-specific functional decline in patients with *FTSJ1* mutations (8–10). However, whether such brain-specific tRNA degradation upon loss of a tRNA modification can be regarded as a general mechanism underlying brain tRNA modopathy remains unclear.

**Figure 1.**
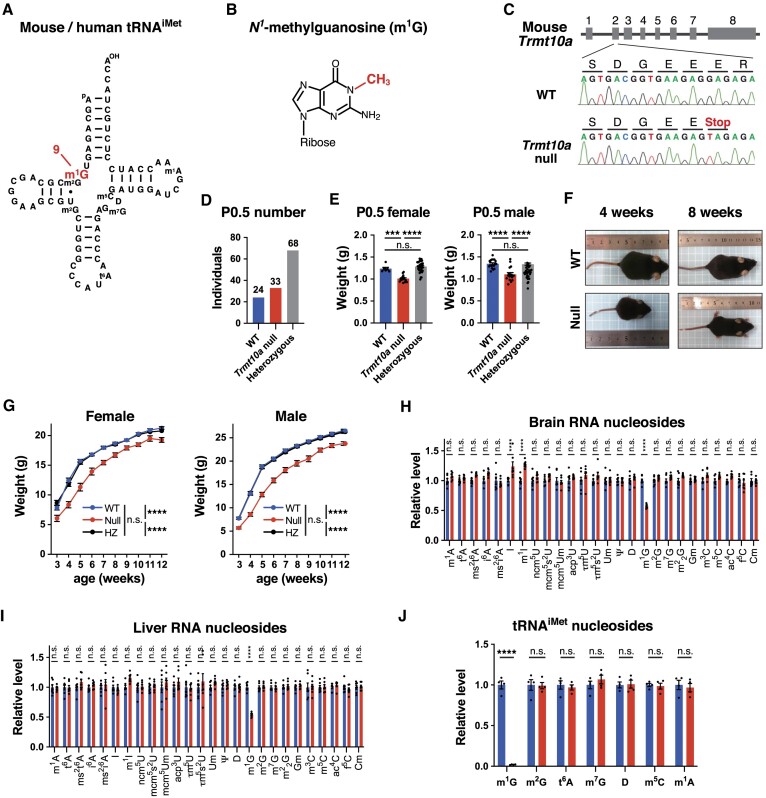
Generation of *Trmt10a* null mice and their reduced body weight. (**A**) Secondary structure of the mouse/human cytoplasmic tRNA^iMet^ with modified nucleosides: *N^1^*-methylguanosine (m^1^G), 2-methylguanosine (m^2^G), *N^6^*-threonylcarbamoyladenosine (t^6^A), 7-methylguanosine (m^7^G), dihydrouridine (D), 5-methylcytidine (m^5^C), and *N^1^*-methyladenosine (m^1^A). Mouse and human tRNA^iMet^ have the same sequences and modifications (67,68). (**B**) Chemical structure of m^1^G. (**C**) *Trmt10a* alleles of WT and *Trmt10a* null mice. The G-to-T mutation generates a premature termination codon in *Trmt10a* gene exon 2 (E29Stop mutation), mimicking a human patient with a homozygotic E27Stop mutation in *TRMT10A* exon 2. Sanger sequence results of the generated mice are shown. (**D**) Number of post-natal day 0.5 (P0.5) pups born after crossing parental heterozygous mice. (**E**) Body weight of WT, *Trmt10a* null, and heterozygous P0.5 pups. *n* = 6 WT females, *n* = 18 WT males, *n* = 14 *Trmt10a* null females, *n* = 17 *Trmt10a* null males, *n* = 40 heterozygous females, and *n* = 29 heterozygous males. ****P < 0.0001, ***P < 0.001 by one-way ANOVA. (**F**) Representative pictures of WT and *Trmt10a* null males at indicated ages. (**G**) Body weight of WT, *Trmt10a* null, and heterozygous mice. Each dot represents the means ± s.e.m. of ≥19 mice. ****P < 0.0001, n.s., not significant by two-way ANOVA. (H, I) LC–MS analysis of total RNA nucleosides generated by nuclease P1 digestion of total RNA from brain (**H**) and liver (**I**) tissue from adult (14–17-week-old) male mice. The peak area of each tRNA-related modified nucleoside was normalized against the uridine peak area in the same sample. The abbreviations for the modified nucleosides not listed in the legend to Figure 1A are listed in Supplementary Table S4. Values are expressed relative to the mean value of the WT samples. Data are presented as the mean ± s.e.m. (*n* = 7 mice each). ****P < 0.001 or n.s. (two-way ANOVA followed by Sidak's multiple comparisons test). (**J**) LC–MS analysis of tRNA^iMet^ nucleosides generated by nuclease digestion of tRNA^iMet^ purified from total RNA extracted from the liver of adult (15–17-week-old) male mice using a biotinylated oligo DNA probe. The peak area of each modified nucleoside was normalized against the uridine peak area in the same sample.

In eukaryotes, the *N^1^*-methylguanosine (m^1^G) modification is present at position 9 in cytoplasmic tRNAs (Figure 1A, B) (3). In human cells, m^1^G9 exists in approximately 40% of all cytoplasmic tRNA species. The m^1^G9 modification is present in most human tRNAs having a G at position 9; the exceptions are tRNA^Gly^, tRNA^Leu^, tRNA^Sec^, and tRNA^Ser^ (Supplementary Table S1) (11–13). In higher eukaryotes, tRNA methyltransferase 10A (TRMT10A) is responsible for m^1^G9 modification of various cytoplasmic tRNAs (14,15). TRMT10A is crucial for human health, and homozygotic mutations in the *TRMT10A* gene is associated with microcephaly, intellectual disability, diabetes, and short stature (16–24).

Some molecular and cellular roles of TRMT10A have been identified using cultured cells. *TRMT10A* knockout in a human haploid cell line decreases the initiator methionine tRNA (tRNA^iMet^) steady-state level (15). Furthermore, lymphoblasts and β-like cells derived from *TRMT10A*-deficient patients show some fragmentation of tRNA^Gln^, which promotes apoptosis (14). In addition to tRNA methylation, TRMT10A is also involved in promoting the degradation of a subset of mRNAs by binding to and supporting the Fat mass and obesity-associated (FTO) protein (25), which is a demethylase for mRNA *N^6^*-methyladenosine (m^6^A) and *N^6^*,2′-*O*-dimethyladenosine (m^6^Am) modifications that promote mRNA decay and/or translation (26,27). However, despite these advances, two fundamental questions remain unsolved: (i) what is the potential role of tRNA m^1^G9 modifications in translation? and (ii) how does TRMT10A dysfunction impair brain function?

Here, we generated *Trmt10a* null mice and performed comprehensive analyses using biochemical, molecular, cellular, physiological, and behavioral approaches. We found that loss of TRMT10A decreased steady-state levels of tRNA^iMet^ and tRNA^Gln^ in various mouse tissues, thereby reducing translation of the corresponding codons, and in particular perturbing translation of mRNAs related to neuronal structures. Furthermore, TRMT10A dysfunction impaired synaptic structure, synaptic plasticity, and memory. These data provide insight into the molecular pathogenesis of a brain tRNA modopathy, and demonstrate the importance of a universal tRNA modification for translation of specific codons.

## Materials and methods

### Animals

A constitutive *Trmt10a* null mouse line was generated by the introduction of the Cas9 protein (Nippon Gene, Tokyo, Japan), trans-activating CRISPR RNA (tracrRNA, FASMAC, Atsugi, Japan), synthetic CRISPR RNA (crRNA, FASMAC), and single-stranded oligodeoxynucleotides (ssODN) into C57BL/6N fertilized eggs by electroporation. The synthetic crRNAs were designed according to the sequence GGGTACGAGTGACGGTGAAG(AGG) of the *Trmt10a* exon 2. The ssODN 5′-ATTGGCTCAGCCCCTGCATCCACTCTAGGCTCCTGTCTCTACTCTTCACCGTCACTCGTACCCAGCTTTTCCTCCACATTAGTAGACTCGAT-3′ was used as a homologous recombination template. The electroporation solution contained 10 μM tracrRNA, 10 μM synthetic crRNA, 0.1 μg/μl Cas9 protein, and 1 μg per μl ssODN in Opti-MEM I Reduced Serum Medium (Thermo Fisher Scientific, Waltham, USA). Electroporation was performed using the Super Electroporator NEPA21 (NEPA GENE, Ichikawa, Japan) on glass microslides with round wire electrodes (1.0 mm gap). Four steps of square pulses were applied (step 1: 3 ms poring pulses three times with 97 ms intervals at 30 V; step 2: 3 ms polarity-charged poring pulses three times with 97 ms intervals at 30 V; step 3: 50 ms transfer pulses five times with 50 ms intervals at 4 V with 40% decay of voltage per pulse; step 4: 50 ms polarity-changed transfer pulses five times with 50 ms intervals at 4 V with 40% decay of voltage per pulse). Backcrossings were performed using C57BL/6N mice.

Mice were housed at 25°C in a 12-h light and 12-h dark cycle. After sacrificing, the tissues were dissected, immediately flash-frozen in liquid nitrogen, and stored at −80°C until the biochemical experiments were conducted. All animal procedures were approved by the Animal Ethics Committee of Kumamoto University (Approval ID: A27-037R1, A2019-1078 R2, A2021-012R2, and A2023-011).

### Cell culture

HEK293FT cells were grown in Dulbecco's modified Eagle's medium (Thermo Fisher Scientific) supplemented with 10% fetal bovine serum (FBS), at 37°C under a humidified atmosphere with 5% carbon dioxide (CO_2_).

### Construction of *TRMT10A* knockout HEK293FT cells

*TRMT10A* knockout cells were generated using the CRISPR/Cas9 system, as described previously (28). Briefly, sense and antisense oligonucleotides encoding a single guide RNA (sgRNA; Supplementary Table S2) were selected from the human GECKO v2 library (29). Sense and antisense oligonucleotides were annealed and cloned into the BsmBI site of the lentiCRISPR v2 plasmid (Addgene #52961). Lentiviruses were generated using the sgRNA-sequence-containing lentiCRISPR v2 plasmid, psPAX2 (Addgene #12260), and pMD2.G (Addgene #12259), and HEK293FT cells were transduced with the generated viruses. After puromycin selection of the transduced cells, single clones were acquired by diluting the cells in 96-well plates.

### Genotyping

Genomic DNA was extracted from the tail end of post-natal day 0.5 (P0.5) or 4-week-old mice. Approximately 50 ng of genomic DNA were subjected to polymerase chain reaction (PCR) to amplify the region surrounding the mutation site using KOD FX DNA polymerase (TOYOBO Life Science, Tokyo, Japan), followed by agarose gel excision of the PCR product and Sanger sequencing using the PCR forward primer. HEK293FT cells were genotyped by PCR amplification surrounding the CRISPR-target region, agarose gel excision of the PCR product, and Sanger sequencing. The primers are listed in Supplementary Table S2.

### Hormone measurements

Blood was collected from the mouse tail and allowed to clot at room temperature for 30 min, followed by centrifugation at 2000 × g for 15 min at 4°C. The serum was collected and stored at -80°C until use. Growth hormone (GH), insulin-like growth factor I (IGF-1), thyroid-stimulating hormone (TSH), and adrenocorticotrophic hormone (ACTH) levels in serum were measured using a Rat/mouse Growth Hormone ELISA Kit (Merck Millipore, Billerica, USA), a Quantikine ELISA Mouse/Rat IGF-I/IGF-1 (R&D Systems, Minneapolis, USA), a Mouse TSH ELISA Kit (Abbexa, Cambridge, United Kingdom), and a Mouse/rat ACTH ELISA Kit (Abcam, Cambridge, United Kingdom), respectively, according to the manufacturers’ protocols.

### RNA extraction

The mouse brain (left or right half), liver (a quarter of the whole liver), heart, and kidney were removed from −80°C storage and immediately placed into 3 ml of TRI Reagent (MRC, Cincinnati, USA) and homogenized using TissueRuptor (Qiagen, Hilden, Germany). The tissue lysate in the TRI Reagent was then centrifuged at 12 000 × g for 10 min, and the supernatant was used for total RNA extraction according to the manufacturer's protocol. For HEK293FT cells, the cells in 10-cm dishes were briefly washed with phosphate-buffered saline (PBS) and lysed in 1 ml of TRI Reagent, followed by total RNA extraction according to the manufacturer's protocol.

### RNA nucleoside mass spectrometry

RNA nucleoside mass spectrometry was performed as previously described (30,31). Briefly, a 25-μl solution containing total RNA (3 μg for LCMS-8050 or 1 μg for LCMS-8060), 20 mM Hepes–KOH (pH 7.6), 2 units of Nuclease P1 (Fujifilm, Tokyo, Japan), and 0.25 units of bacterial alkaline phosphatase (Takara, Kusatsu, Japan) was incubated at 37°C for 3 h. Subsequently, 3 μl of the nucleoside solution was injected into the LCMS-8050 or LCMS-8060 system (Shimadzu, Kyoto, Japan). The nucleosides were first separated by an Inertsil ODS-3 column (GL Science, Tokyo, Japan) using a mobile phase that continuously changed from 100% of solution A (5 mM ammonium acetate in water, pH 5.3) to 100% of solution B (60% acetonitrile in water) in 17 min at a flow rate of 0.4 ml min^−1^, followed by electrospray ionization and triple quadrupole mass spectrometry in multiple reaction monitoring mode.

### Isolation of tRNA^iMet^ from total RNA

tRNA^iMet^ was isolated from total RNA as described previously (28). Briefly, 1.5 nmol of a 3′ biotinylated DNA probe (Supplementary Table S2) was bound to streptavidin beads (GE Healthcare, Chicago, USA) for 1 h at room temperature in binding buffer (100 mM NaCl, 10 mM Hepes–KOH pH 7.6, and 5 mM EDTA), followed by washing with binding buffer and hybridization buffer (1200 mM NaCl, 30 mM Hepes–KOH pH 7.6 and 7.5 mM EDTA). The probe-bound beads were mixed with 100 μg of total RNA in hybridization buffer and then incubated at 65°C for 1 h with occasional agitation. The beads were washed ten times with wash buffer (600 mM NaCl, 30 mM Hepes–KOH pH 7.6, and 7.5 mM EDTA) at 65°C and then eluted with elution buffer (20 mM NaCl, 0.5 mM Hepes–KOH pH 7.6, and 0.25 mM EDTA) at 65°C. The eluate was subjected to 7 M urea/Tris–borate–EDTA (TBE)/10% polyacrylamide gel electrophoresis (PAGE), followed by SYBR Gold staining and excision of the tRNA band from the gel.

### tRNA sequencing

Total tRNA sequencing (tRNA-seq) was performed as described previously (32). Briefly, 6 μg of total RNA was resolved by 7 M urea/TBE/10% polyacrylamide gel, and total tRNA (60–100 nt) was collected by gel excision. Total tRNA was demethylated using *E. coli*-derived AlkB (Addgene #79050) to enable reverse transcription (12). In brief, total tRNA was incubated in 45 mM Tris–HCl (pH 8), 0.9 mM α-ketoglutaric acid, 1.8 mM ascorbic acid, 67 μM (NH_4_)_2_Fe(SO_4_)_2_, and 2.5 μM AlkB at 37°C for 2 h. Demethylation was confirmed by simultaneously performing yeast tRNA demethylation and RNA nucleoside mass spectrometry analysis as a positive control. tRNA and Air Adenylated Linker A (BIOO, Barcelona, Spain) were incubated at 60°C for 3 min to denature and then ligated using T4 RNA ligase 2 truncated (NEB, Ipswich, USA), according to the manufacturer's protocol. The 3′adopter-ligated tRNA was urea gel-excised and reverse-transcribed using Rever Tra Ace (Toyobo) and the primer listed in Supplementary Table S2. cDNA was gel-excised and circularized using CircLigase II (Lucigen, Miami, USA) according to the manufacturer's protocol. cDNA amplification and barcode sequence addition were performed by PCR using the primers listed in Supplementary Table S2 and PrimeSTAR Max (TAKARA). The PCR product was purified by gel excision after native TBE/10% PAGE, and then subjected to quantitative PCR using TB Green (TAKARA), Rotor Gene (Qiagen), KAPA Library Quant (Illumina, San Diego, USA), and the primers listed in Supplementary Table S2. The cDNA library was sequenced using MiSeq (Illumina). Galaxy (33) was used for adaptor trimming of fastq files, bowtie2 mapping of the obtained sequences to the mouse tRNA sequences retrieved from the Genomic tRNA Database (11), conversion to Sequence Alignment/Map (SAM) files, and counting using the ‘cut column’ and ‘group’ functions.

### Northern blot analysis

Total RNA (1.5 μg) was separated using electrophoresis in 7 M urea/TBE/10% polyacrylamide gel at 150 V. The gel was then stained with SYBR Gold (Invitrogen, Carlsbad, USA) to check RNA quality and then transferred to a nylon membrane (Merck Millipore, Billerica, USA) in 1 × TBE using a wet transfer blotting system (Bio-Rad, Hercules, USA) on ice at 50 V for 80 min. Membranes were dried and crosslinked with ultraviolet light at 1200 × 100 μJ cm^−2^ using HL-2000 Hybrilinker (Funakoshi, Tokyo, Japan) and incubated in prehybridization buffer (6 × saline sodium citrate (SSC), 0.1% sodium dodecyl sulfate (SDS) and 1 × Denhardt's solution) at 42°C for 1 h. The membranes were then hybridized with digoxigenin (DIG)-labeled (Roche, Basel, Switzerland) probe DNA in hybridization buffer (900 mM NaCl, 90 mM Tris–HCl pH 8, 6 mM EDTA and 0.3% SDS) overnight at 50°C. The membranes were washed with 1 × SSC, blocked using DIG wash and block buffer set (Roche), and probed with anti-DIG alkaline phosphatase Fab fragments (Roche) and CDP-Star (Roche). Images were acquired using ImageQuant (GE Healthcare). Aminoacyl-tRNA analysis was performed as described previously (34,35). Briefly, after the total RNA pellet was collected from the tissues using TRI Reagent and 2-propanol precipitation, the RNA pellet was rinsed with 75% ethanol/10 mM NaOAc (pH 5.0)/1 mM EDTA and dissolved in 10 mM NaOAc (pH 5.0)/1 mM EDTA. The aminoacylation level was monitored using acidic PAGE, followed by conventional northern blot, using the same DIG-labeled probes as conventional northern blot. Probe DNA sequences are listed in Supplementary Table S2.

### Ribosome profiling

Ribosome profiling (36) was performed as described previously (8), with several modifications. Briefly, the mouse brain half stored at − 80°C was transferred into ice-cold polysome buffer (32) and homogenized using TissueRuptor (Qiagen). RNase I (Thermo Fisher Scientific) was added at a ratio of 1 μl per 30 μg of RNA (estimated by measuring the optical density (OD) using a Biospectrometer; Eppendorf, Hamburg, Germany) and incubated for 45 min on ice. This was followed by addition of 10 μl of RNase inhibitor (Nacalai, Kyoto, Japan) and centrifuging at 100 000 rpm for 2 h using a TLA110 rotor (Beckman Coulter, Brea, USA) to pellet the ribosomes through a sucrose cushion (1 M sucrose, 20 mM Tris–HCl pH 7.5, 150 mM NaCl, 5 mM MgCl_2_, 1 mM DTT and 100 μg/ml cycloheximide). RNA was extracted from the pellet using the TRI Reagent (MRC). Library preparation was performed as described for tRNA-seq, except that T4 PNK (NEB) treatment in the absence of ATP was performed instead of AlkB treatment. The library was sequenced using NovaSeq (Illumina). Galaxy (33) was used to filter fastq files by quality, followed by removal of the adaptor sequence, alignment by HISAT2, counting of reads by HTSeq-count, and WT versus null comparison by DESeq2. Gene ontology analysis was performed using Gene Set Enrichment Analysis website software (37). For *Atf4* and *Atf5* ribosome footprint analysis, the adaptor sequence was removed and the fastq files were aligned against mouse rRNAs. The rRNA-removed reads were aligned against *Atf4*, *Atf5* and *actin* cDNA sequences, and the aligned reads were counted using the bowtie2, BAM-to-SAM, cut, and group functions in Galaxy (33) and then visualized using the Integrative Genomics Viewer (38).

### Western blot analysis

Western blot was performed as previously described (39). Briefly, the left or right half of the mouse brain stored at − 80°C was transferred into 1 ml of lysis buffer (150 mM NaCl, 100 mM Tris–HCl pH 8, 0.5% NP-40, a protease inhibitor cocktail [Roche], and a phosphatase inhibitor cocktail [Nacalai]), homogenized using TissueRuptor (Qiagen), and sonicated for 10 s. The protein concentration was determined using a bicinchoninic acid (BCA) protein assay kit (Thermo Fisher Scientific). Samples were electrophoresed in SDS polyacrylamide gel and transferred to an Immobilon-P membrane (Merck Millipore, Billerica, USA). The membrane was blocked with 5% skim milk in TBST buffer (150 mM NaCl, 25 mM Tris–HCl pH 7.4, 2.7 mM KCl and 0.05% Tween-20) and probed for respective proteins using the primary antibodies diluted in 5% skim milk in TBST buffer at 4°C overnight. The membrane was washed in TBST, probed using the secondary antibody at room temperature for 1 h, and washed again in TBST. The signals were detected using an ECL Prime Western Blotting Detection Reagent (GE Healthcare) and an Image Quant 400 imager (GE Healthcare). The antibodies and their dilutions are listed in the Supplementary Table S3.

### ^35^S-methionine labeling of nascent proteins

Pulse-labeling of nascent proteins was performed as previously performed (28,40), with several modifications. Briefly, 1 day before radiolabeling, 1.2 × 10^6^ wild-type (WT) or *TRMT10A* knockout (KO) HEK293FT cells were seeded into 6-cm dishes. The next day, the cells were briefly washed using 37°C DMEM without Met, Cys and Gln (Thermo Fisher Scientific). A total of 3 ml of 37°C preincubation medium (DMEM without Met, Cys, or Gln, supplemented with 2% FBS, 2 mM Gln and 0.2 mM Cys) was added to the cells, and the cells were incubated in a 37°C CO_2_ incubator for 15 min. The medium was then exchanged with 2 ml of incubation medium (2 ml of preincubation medium containing 5.92 MBq of ^35^S-labeled methionine [PerkinElmer]), and the cells were incubated in a 37°C CO_2_ incubator for 40 min. Subsequently, the medium was removed, and the cells were washed with PBS and collected using trypsin and DMEM containing 10% FBS. Cells were lysed and the protein concentration was measured using a BCA Protein Assay Kit (Thermo Fisher Scientific). Then, 24 μg of total protein were run on Tricine PAGE gel (NOVEX, Carlsbad, USA). The gel was stained using Coomassie brilliant blue staining solution (Bio-Rad), dried on a gel dryer, and photographed, and the radiation image was acquired using an imaging plate and imager (Fujifilm).

### Pancreas, liver and kidney histology

Under deep anesthesia by intraperitoneal injection of pentobarbital, mice were fixed by perfusion of 10% formaldehyde (Wako) in PBS. Subsequently, tissues were embedded in paraffin, and hematoxylin and eosin (H&E) staining was performed for the pancreas and liver by K. I. Stainer, Inc. (Kumamoto, Japan). Periodic acid-Schiff (PAS) staining was performed on the kidney sections (2-μm thick) according to standard protocols. The stained samples were observed under a light microscope (Olympus, Tokyo, Japan).

### Glucose tolerance test

The glucose tolerance test was performed as performed previously (41). Briefly, mice were fasted for 14 h (9 pm to 11 am) in cages with new bedding, followed by intraperitoneal injection of glucose (1 g/kg). Blood glucose concentration was measured using an ACCU-CHEK glucometer (Roche), before (0 min) and 15, 30, 45, 60, 75 and 90 min after glucose injection.

### Serum and urine biochemical measurements

Under deep anesthesia by intraperitoneal injection of pentobarbital, mouse blood was collected from the vena cava using a 26-gauge needle and a syringe. The blood was allowed to clot at room temperature for approximately 1 h, followed by centrifugation at 1800 × g. The supernatant was then collected as serum samples. The total bilirubin and creatinine concentrations of the mouse serum were measured by enzymatic assays. Serum urea nitrogen concentration was measured using the urease-glutamate dehydrogenase method. Serum albumin concentration was measured using the bromocresol green method. Serum amylase, aspartate aminotransferase (AST), and alanine transaminase (ALT) concentrations were measured by the Japanese Committee for Clinical Laboratory Standards-transferable methods. The serum biochemical measurements were performed by Oriental Yeast Co. Ltd. (Tokyo, Japan). Urine was collected by keeping each mouse in a metabolic cage for 24 h. Mouse urine sodium and potassium ion concentrations were measured using ion-selective electrode methods. Urine creatinine concentration was measured by enzymatic assay. The urine biochemical measurements were performed by SRL, Inc. (Tokyo, Japan).

### Immunofluorescence analysis

Under deep anesthesia by inhalation of isoflurane, mice were fixed by perfusion through the ascending aorta with 4% paraformaldehyde (PFA) and 0.1% glutaraldehyde in 0.1 M phosphate buffer (PB). Serial 40-μm thick coronal sections containing the hippocampus and somatosensory cortex were cut using a vibrating microtome (DTK-3000, Dosaka). Sections were processed for triple-label immunofluorescence using a mixture of goat antibody against vesicular glutamate transporter 1 (vGluT1, Frontier Institute, Ishikari, Japan; dilution 1:500), guinea pig antibody against vGluT2 (Frontier Institute, dilution 1:500), and rabbit antibody against glutamic acid decarboxylase 65/67 (GAD65/67, Sigma, dilution 1:5000). Signals were visualized using a combination of secondary antibodies consisting of biotinylated anti-goat immunoglobulin G (IgG; Jackson ImmunoResearch, Baltimore, USA), followed by streptavidin-Alexa 647 (Jackson ImmunoResearch), Alexa 488-conjugated anti-guinea pig IgG (Jackson ImmunoResearch), and rhodamine red-conjugated anti-rabbit IgG (Jackson ImmunoResearch); all secondary antibodies are those raised in donkey. After embedding in Vectashield (Vector Labs, Newark, USA), images were acquired using a confocal laser scanning light microscope (CLSM, Nikon C2). The thickness of the cerebral cortex was measured at the barrel field of the primary somatosensory area (S1BF). Eight coronal sections of the S1BF in each mouse were measured and averaged.

### Quantification of axonal bouton size

Because the size of the brains of null mice was smaller than that of WT littermates, the rostrocaudal position of the region in the hippocampus used for morphological analysis was objectively determined using internal landmarks rather than a brain atlas; coronal sections at the same distance from the bregma were found to differ in neuroanatomical profiles between the WT and null mice. The two landmarks along the rostrocaudal axis of the hippocampus were selected as follows: one was the rostral end of the dorsal hippocampus and the other was the section from which the dorsal subiculum appeared and extended caudoventrally. According to the standard brain atlas (42), these two landmarks in WT mice corresponded to the levels of −0.95 and −2.45 mm from the bregma, respectively. Sections located at the mid-rostrocaudal level between these landmarks were selected for analysis. Next, the mid-proximodistal position of the CA1 region in each section was selected. Finally, the mid-apicobasal position in the stratum radiatum was selected for analysis.

Quantification of axonal bouton size was performed as described previously (43,44). High-resolution CLSM images of vGluT1-positive boutons were acquired using a 60× objective with a zoom factor of 2.0 in a 1024 × 1024-pixel frame, where 1 pixel corresponded to 0.10358 μm. Conditions of the CLSM settings, such as laser power and gain of photon multiplier, were kept constant during the acquisition of bouton images in both the WT and *Trmt10a* null brains. The single optical slice images were processed for morphological quantification using a series of automatic commands included in the image analysis software ImageJ (National Institute of Health, Bethesda, USA) as follows: background signals were subtracted using a ‘Rolling-ball’ command, signals were thresholded at a constant gray level to generate binary images, and the binary images were then filtered using the ‘Open’ command, which performs an erosion operation followed by dilatation to smooth objects and remove isolated pixels. Following these procedures, the areas of the individual boutons were measured using the ‘Analyze Particle’ command under the condition that objects larger than 100 pixels (which corresponded to profiles of adhesion of multiple boutons) were excluded from the measurements.

### Transmission electron microscopy

Under deep anesthesia by inhalation of isoflurane, mice were fixed by perfusion through the ascending aorta with 2% PFA and 2.5% glutaraldehyde in 0.1 M PB. Serial 40-μm thick coronal sections containing the hippocampus were cut using a vibrating microtome (DTK-3000, Dosaka). After washing in PBS, the tissues were post-fixed with 1% OsO_4_ in PB on ice for 1 h. The tissues were stained en bloc with 1.5% uranyl acetate at 4°C for 1 h, dehydrated in ethanol, infiltrated in propylene oxide, and finally flat-embedded between a coverslip and a slide using epoxy resin. After hardening of the resin and removal of the coverslips and slides, the region of interest was defined using the same method as described above for measuring the size of the axonal boutons in the CLSM images. The selected regions for electron microscopy (EM) analysis were excised from the resin-embedded sections and re-embedded on top of a resin cylinder. Ultrathin sections (65 nm in thickness) were cut using an EM UC7 ultramicrotome (Leica, Wetzlar, Germany) and stained with 1.5% uranium acetate and lead citrate. The images were acquired at 80 kV using an HT7700 transmission electron microscope (Hitachi, Tokyo, Japan). Random section images were used to measure the length of post-synaptic density (PSD) with the ImageJ software.

### Behavioral tests

Ten- to 13-week-old mice were handled for at least 3 days. Mice were then subjected to the open-field test, Barnes maze, and rotarod test, with approximately 1-week intervals between each test. For the open-field test, a gray 60 × 60 cm chamber was used. Mice were placed in the center of the chamber, and exploratory behavior was recorded for 30 min using a video camera above the chamber. Video software (Actimetrics, Wilmette, USA) was used to define a five-by-five grid, and the central three-by-three grid was defined as the center area. Total distance traveled in the entire area of the chamber, and time spent in the center or outside areas was quantified.

The Barnes maze was performed as described previously (45), with several modifications. The Barnes maze consisted of a circular platform (diameter: 91 cm) elevated 100 cm above the floor, with equally spaced holes (diameter: 5 cm) around the perimeter. A black box was mounted underneath one of the holes on the platform to enable the mouse to escape from the open platform surface. Visual cues were placed around the maze. The black escape box was maintained at a fixed location for each mouse throughout the experiment period but at a different location for each mouse. A video camera was placed above the platform to record the movements of the mice. On the trial day, the mouse was placed in a dark start chamber in the center of the platform. After 10 s, the start chamber was lifted, and the movement of the mouse was recorded for 3 min. If the mouse was unable to locate the black box within 3 min, the trial was terminated by gently guiding the mouse toward the black box. For 4 consecutive days, the mice received four trials per day with intervals of 20 min. The time to reach the target black box in the first trial on each of the 4 trial days was measured.

The rotarod test was performed as described previously (46). A rotating rod was set with a start speed of 4 rpm. The mouse was held by the tail and placed on the rotating rod in the direction that required it to walk forward to stay upright. The mouse was allowed to walk on the rod at 4 rpm for 10 s, followed by continuous rod acceleration at 20 rpm/min. The time taken for the mouse to fall from the rod was recorded.

### Electrophysiology

To evaluate neuronal plasticity, the hippocampal slices were prepared as described previously (47). Briefly, the brains were quickly removed from ether-anesthetized mice and immediately chilled in ice-cold, oxygenated artificial cerebrospinal fluid containing 124 mM NaCl, 5 mM KCl, 26 mM NaHCO_3_, 2 mM CaCl_2_, 2 mM MgSO_4_, 1.25 mM NaH_2_PO_4_, and 10 mM d-glucose. The hippocampal slices were cut into sagittal slices with a thickness of 400 μm using a 7000smz-2 vibratome (Campden Instruments, Loughborough, England) and transferred to a recording chamber, where they were left to recover for at least 1 h at room temperature (24 ± 2°C) before recording. A concentric bipolar stimulating electrode was inserted into the stratum radiatum of the CA1 to stimulate the Schaffer collateral pathway. High-frequency stimulation (HFS) at 100 Hz for a duration of 1 s was applied three times with a 20-s interval. Traces were obtained and analyzed using SutterPatch version 2.2 (Sutter Instrument, Novato, USA).

### Statistical analysis

All numerical data were analyzed using the GraphPad Prism 9 or 10 software. Welch's *t*-test or Mann–Whitney test was used to assess differences between the two groups. When comparing more than two groups, an analysis of variance (ANOVA) was used. A two-tailed *P*-value of 0.05 was considered significant. Data are presented as mean ± standard error of the mean (s.e.m.).

## Results

### *Trmt10a* null mice show reduced body weight

To explore the molecular and physiological roles of TRMT10A-mediated m^1^G9 modification, we generated whole-body *Trmt10a* null mice by introducing a point mutation into the endogenous *Trmt10a* gene to mimic a mutation found in *TRMT10A*-deficient patients (23). Specifically, a G-to-T mutation was introduced to generate a stop codon in the endogenous *Trmt10a* exon two, which produced an E29stop mutation in the TRMT10A protein (Figure 1C). After five generations of backcrossing, heterozygous males and females were crossed to generate *Trmt10a* null mice. The WT, *Trmt10a* null, and heterozygous mice were born at an approximately Mendelian ratio (Figure 1D), which suggested that *Trmt10a* deficiency does not fatally impair embryonic development, similar to human patients being born with homozygotic *TRMT10A* null mutations. The *Trmt10a* null mice had lower body weight at post-natal day 0.5 (P0.5) (Figure 1E), as well as during the growth period and adulthood (Figure 1F, G). The lower body weight of the *Trmt10a* null mice mimics that in human patients with defective *TRMT10A*, who typically have short stature (16–24).

To investigate whether the levels of growth-related hormones are lower in *Trmt10a* null mice than in the WT, we measured the concentrations of growth hormone (GH), insulin-like growth factor I (IGF-1), thyroid-stimulating hormone (TSH), of which GH and TSH are produced by the brain pituitary gland. There were no clear differences in the levels of these growth-related hormones between the serum from *Trmt10a* null and WT mice (Supplementary Figure S1A).

We then extracted total RNA from the brain and liver and confirmed that the amount of m^1^G in total RNA from *Trmt10a* null brain and liver tissues had decreased to about 60% of that in WT mice (Figure 1H, I). We postulate that the remaining m^1^G was derived from m^1^G at position 37 of various tRNAs (3). In tRNA^iMet^, m^1^G exists only at position nine (Figure 1A) (3), and was confirmed to be lost from tRNA^iMet^ purified from total RNA extracted from *Trmt10a* null mice, whereas there was no change in the amount of all other tRNA^iMet^ modifications (Figure 1J).

Thus, our *Trmt10a* null mice may serve as a suitable animal model to study the physiological functions of TRMT10A.

### A universal decrease in the steady-state levels of tRNA^iMet^ and tRNA^Gln^ in various mouse tissues and a human cell line lacking TRMT10A

**Figure 2.**
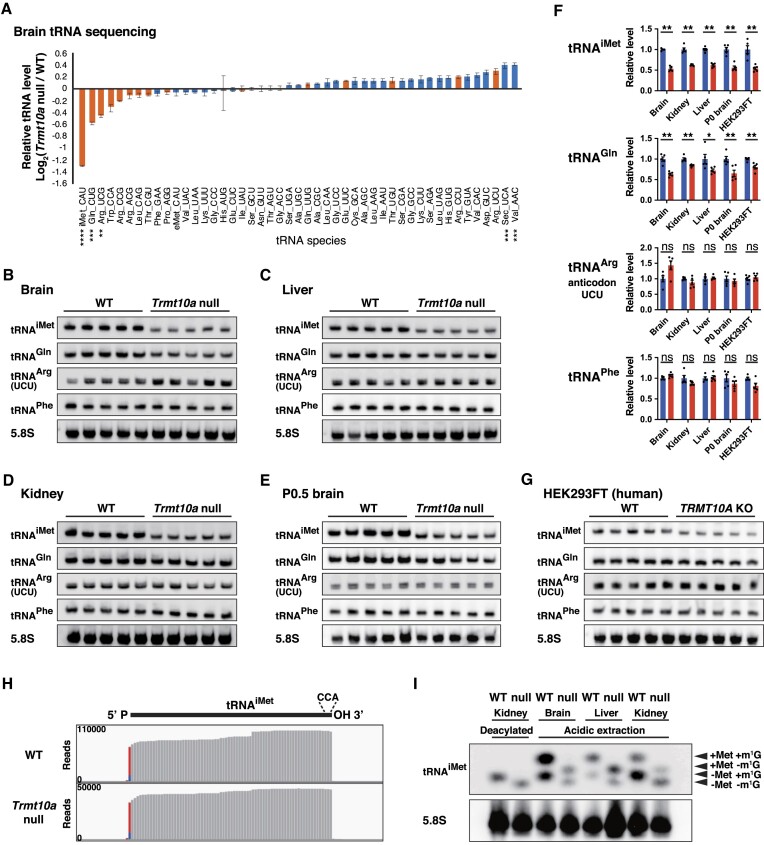
Universal decrease in tRNA^iMet^ and tRNA^Gln^ levels in mouse tissues and a human cell line. (**A**) Relative abundance of individual tRNA species in the 15-week-old male *Trmt10a* null mouse brains in comparison to WT brains. Means ± s.e.m. from *n* = 4 WT and *n* = 4 *Trmt10a* null mouse brains. ****P < 0.0001, ***P < 0.001, **P < 0.01 by two-way ANOVA followed by Sidak's test. (B–E) Northern blot analysis of tRNA^iMet^, tRNA^Gln^, tRNA^Arg(UCU)^, tRNA^Phe^ and 5.8S rRNA from 15-week-old (adult) male mouse brains (**B**), adult livers (**C**), adult kidneys (**D**) and P0.5 pup brains (**E**). Each lane derives from one mouse (*n* = 5 WT and *n* = 5 *Trmt10a* null). Note that although the tRNA^Gln^ probes were designed to recognize tRNA^Gln(CUG)^, the probe might also bind to tRNA^Gln(UUG)^ due to the sequences having only a single nucleotide mismatch. Long exposure images of the whole membrane northern blots of brain tRNA^iMet^ and tRNA^Gln^ are shown in Supplementary Figure S2A and S2C. (**F**) Quantification of tRNA levels in northern blots in B–E and G. tRNA was normalized by 5.8S rRNA. Means ± s.e.m. from *n* = 5 WT and *n* = *Trmt10a* null mouse tissues or HEK293FT cells. **P < 0.01, *P < 0.05 by Mann–Whitney test. (**G**) Northern blot analysis of tRNA^iMet^, tRNA^Gln^, tRNA^Arg(UCU)^, tRNA^Phe^ and 5.8S rRNA from WT and *TRMT10A* KO HEK293FT cells. (**H**) Reads mapped to tRNA^iMet^ in the brain tRNA-seq in Figure 2A. One extra nucleotide in the 5′ end (shown in red) likely derives from the terminal transferase activity of the reverse transcriptase. (**I**) Aminoacyl-tRNA northern blot of tRNA^iMet^ and 5.8S rRNA (loading control). The two left samples are deacylated control samples. The samples acquired by acidic extraction reflect the physiological aminoacylation status. tRNAs from *n* = 3 WT and *n* = 3 *Trmt10a* null 15–16-week-old male mice were analyzed. A representative image from a WT and *a Trmt10a* null mouse is shown. Results obtained from additional mice, as well as statistical analysis of the quantified ratio of aminoacylated and non-aminoacylated tRNAs, are shown in Supplementary Figures S2E and S2F.

To explore the global effect of TRMT10A in all tRNAs, we performed tRNA-seq of *Trmt10a* null and WT mouse brains. We observed reductions in several m^1^G9-containing tRNAs. In particular, the initiator methionine tRNA (tRNA^iMet^) and the tRNA^Gln^ with the CUG anticodon (tRNA^Gln(CUG)^) showed the largest decreases in *Trmt10a* null brains (Figure 2A). Interestingly, cytoplasmic tRNAs reported to harbor the m^1^G9 modification in human cells (orange bars in Figure 2A) (12) were concentrated within tRNAs that showed a decrease in *Trmt10a* null brains, suggesting that m^1^G9 is important for maintaining steady-state levels of several m^1^G9-containing tRNA species. Next, we validated the tRNA-seq results by northern blotting, after which we observed a decrease in tRNA^iMet^ and tRNA^Gln^ levels, and a limited (or no) change in m^1^G9-containing tRNA^Arg(UCU)^ and m^1^G9-unmodified tRNA^Phe^ (Figure 2B, Supplementary Figure S2A–S2D). Similar to brain, we found that tRNA^iMet^ and tRNA^Gln^ levels decreased in the adult liver and kidney (Figure 2C, D). Moreover, these tRNAs were decreased also in newborn P0.5 mouse brains (Figure 2E, F). To test the effect of TRMT10A in human cells, we generated *TRMT10A* knockout HEK293FT cells (Supplementary Figure S3A, S3B), and confirmed that (similar to mouse tissues) levels of tRNA^iMet^ and tRNA^Gln^ decreased (Figure 2G). We did not find a significant change in tRNA^Arg(UCU)^ and tRNA^Phe^ in any of the mouse tissues and human cells (Figure 2B–G). Notably, a similar reduction in tRNA^iMet^ levels was also reported in a *TRMT10A* knockout human haploid cell line (15).

During northern blotting, we noticed that the tRNA^iMet^ bands appeared to shift down slightly upon loss of TRMT10A (Figure 2B–E, G), which is in line with a previous report that used a human *TRMT10A* KO haploid cell line (15). The band shift is not likely due to exoribonucleolytic degradations because our tRNA-seq mapping showed that there was no major difference in the 5′ and 3′ ends of tRNA^iMet^ between WT and *Trmt10a* null brains, including the CCA tail (Figure 2H). In addition, the aminoacyl-tRNA northern blot showed that there was no difference in the tRNA^iMet^ aminoacylation status, at least in the brain (Figure 2I, Supplementary Figure S2E, S2F). In addition, there was no difference in the modification level of tRNA^iMet^, except m^1^G9, upon loss of *Trmt10a* (Figure 1J). Taken together, the data suggest that the band shift of tRNA^iMet^ in the standard northern blots likely derived from the lack of m^1^G9 modification.

Thus, our results show that TRMT10A is universally required to maintain steady-state levels of tRNA^iMet^ and tRNA^Gln^ across various mouse tissues, as well as in a human cell line.

### Lack of an apparent phenotype in pancreas, liver and kidney tissues, and the presence of smaller postsynaptic densities in the hippocampus of the *Trmt10a* null mice

**Figure 3.**
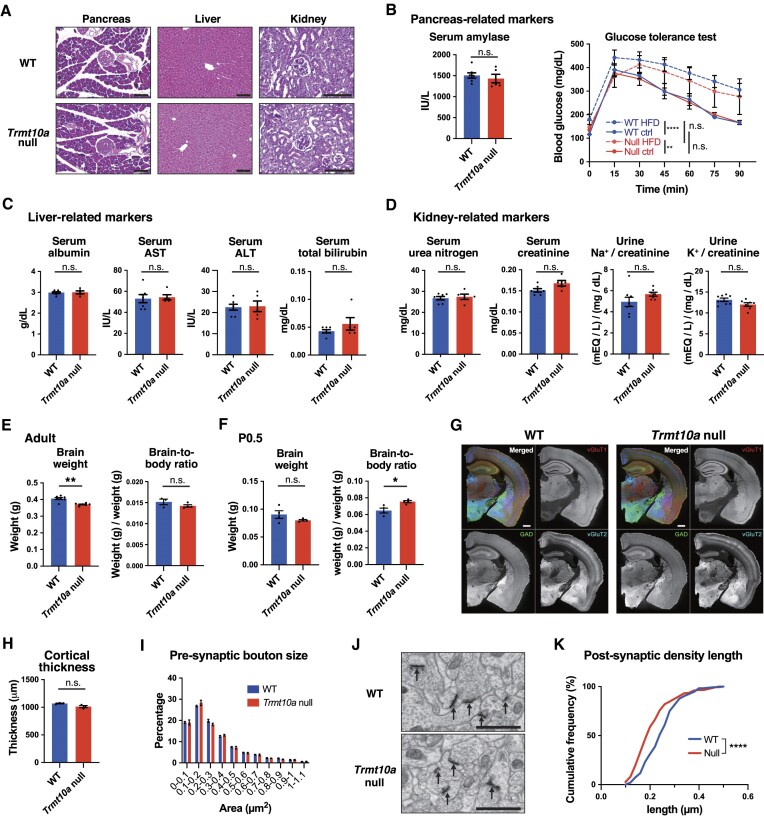
Tissue phenotypes in *Trmt10a* null mice. (**A**) Pancreas, liver, and kidney slices of 8-week-old WT and *Trmt10a* null males. Pancreas and liver samples were subjected to H&E staining and kidney samples to PAS staining. Scale bar, 100 μm. (**B**) Pancreas-related markers. Left, serum amylase levels. Means ± s.e.m. from *n* = 7 WT and *n* = 5 *Trmt10a* null 17-week-old females. Right: glucose tolerance test performed in mice fed with a normal diet (ctrl) or a high-fat diet (HFD). Means ± s.e.m. from *n* = 4 WT fed with the ctrl diet, *n* = 4 *Trmt10a* null fed with the ctrl diet, *n* = 5 WT fed with the HFD and *n* = 4 *Trmt10a* null fed with the HFD, 20-week-old males. ****P < 0.0001, **P < 0.01 by two-way ANOVA. (**C**) Liver-related markers detected from the same serums as in (B). (**D**) Kidney-related markers detected from the same serums as in (B). Urine samples were collected from *n* = 8 WT and *Trmt10a* null 10- to 11-week-old males. Urine Na^+^ and K^+^ were normalized by urine creatinine. (**E**) Adult brain weight (left) and brain-to-body weight ratio (right). Means ± s.e.m. from *n* = 7 WT and *n* = 7 *Trmt10a* null,16- to 20-week-old males. **P < 0.01 by Mann–Whitney test. (**F**) P0.5 pup brain weight (left) and brain-to-body weight ratio (right). *n* = 4 WT and *n* = 4 *Trmt10a* null males. *P < 0.05 by Mann–Whitney test. (**G**) Immunofluorescence of vGluT1, vGluT2, and GAD65/67 in brains of 14-week-old males. Scale bar, 500 μm. (**H**) Cortical thickness. Means ± s.e.m. from *n* = 3 WT and *n* = 3 *Trmt10a* null 14-week-old males. (**I**) Presynaptic bouton size measured by quantification of 3894 boutons in four images from *n* = 2 WT 14-week-old males and 3544 boutons in four images from *n* = 2 *Trmt10a* null 14-week-old males. (**J**) Representative electron microscopic images of hippocampal post-synaptic density (PSD, arrows). Scale bar, 1 μm. (**K**) Cumulative frequency distribution of PSD length from the hippocampus of WT and *Trm10a* null 14-week-old males. We measured 189 synapses (both excitatory and inhibitory synapses) from six WT images and 178 synapses from eight *Trmt10a* null images. ****P < 0.0001 by Kolmogorov-Smirnov test.

Human patients with homozygotic *TRMT10A* mutations typically show short stature, diabetes, and brain-related pathologies (microcephaly and intellectual disability) (16–24). Consistent with this, we observed lower body weight in *Trmt10a* null mice (Figure 1E–G); therefore, we investigated whether *Trmt10a* null mice show similar defects in pancreatic glucose regulation and brain tissue. Surprisingly, and unlike human patients with *TRMT10A* mutations, *Trmt10a* null mice had normal pancreatic tissue (Figure 3A), normal serum amylase levels (a marker of pancreatic injury or inflammation; Figure 3B, left), and normal blood glucose regulation (Figure 3B, right). A high-fat diet weakened blood glucose regulation in *Trmt10a* null mice, but the level of weakening was similar to that in WT mice (Figure 3B, right). Although liver or kidney abnormalities are not common in human patients with *TRMT10A* mutations, we examined the liver and kidneys of *Trmt10a* null mice. Liver tissue sections and measurement of markers of liver protein production and injury (serum albumin, AST, ALT, and bilirubin) did not reveal significant differences between *Trmt10a* null and WT mice (Figure 3A, C). Similarly, the kidney tissue sections, and levels of function markers serum urea nitrogen, serum creatinine, and urine sodium or potassium (normalized against creatinine), did not show major differences (Figure 3A, D).

The weight of adult *Trmt10a* null brains was lower than that of WT brains, yet there was no difference in the brain-to-body weight ratio (Figure 3E). In addition, P0.5 mice did not show a significant reduction in brain weight (Figure 3F). Brain tissue sections were triple-immunolabeled to detect vGLUT1, vGLUT2, and GAD65/67; the former two transporters are markers of excitatory axon terminals originating from cortical and subcortical neurons, respectively, whereas GAD65/67 is a marker of inhibitory neurons (Figure 3G). We observed that the overall morphological appearance and neuronal distribution in brain tissue (Figure 3G), as well as cortical thickness (Figure 3H) and presynaptic bouton size (Figure 3I), did not differ between WT and *Trmt10a* null brains; however, EM observations revealed that postsynaptic density (PSD), an electron-dense region just beneath the postsynaptic membrane showing an accumulation of synapse-related proteins, was smaller in the CA1 region of the hippocampus of *Trmt10a* null brains than in that of WT brains (Figure 3J, K). Considering that reduced PSD size is often associated with brain dysfunctions such as memory impairment (8), we next examined protein synthesis in the brain.

### Ribosome slowdown at the Gln CAG codon and perturbed translation in the *Trmt10a* null brain

**Figure 4.**
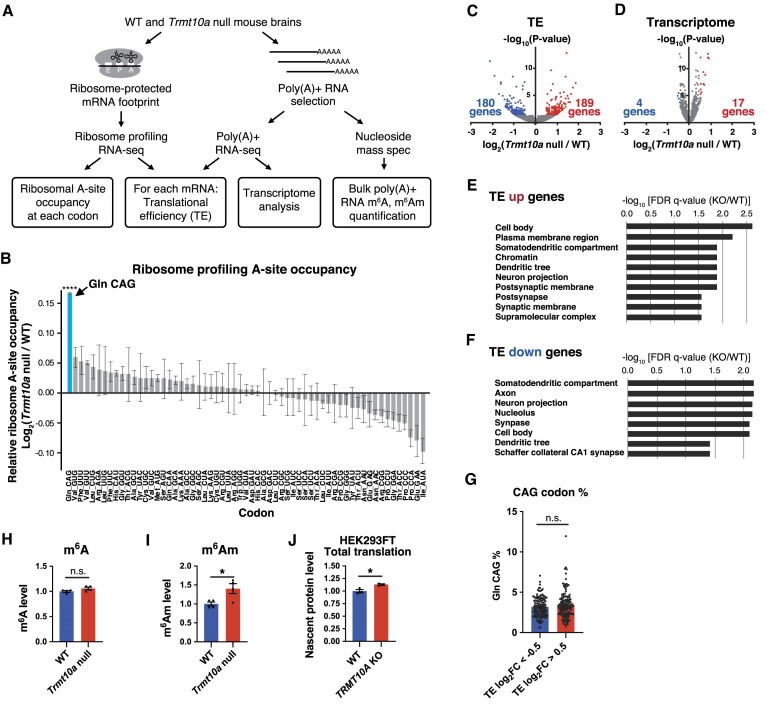
Ribosome slowdown at the Gln CAG codon and perturbed translation in the *Trmt10a* null brain. (**A**) Strategy for ribosome profiling, transcriptome analysis, and bulk poly(A)+ RNA modification analysis. (**B**) Relative ribosome A-site occupancy at each mRNA codon. Means ± s.e.m. from *n* = 3 WT and *n* = 3 *Trmt10a* null 14-week-old male mice. ****P < 0.0001 by Welch's *t*-test. Unindicated codons showed non-significant changes. (**C**) Volcano plot of brain translational efficiency (TE) in WT versus *Trmt10a* null male brains. Genes with *P* < 0.05 and log_2_(fold change null/WT) > 0.5 are plotted in red, and genes with *P* < 0.05 and log_2_(fold change null/WT) < −0.5 are plotted in blue. (**D**) Volcano plot of brain transcriptome in *n* = 5 WT versus *n* = 5 *Trmt10a* null 14-week-old male brains. Genes with *P* < 0.05 and log_2_(fold change null/WT) > 0.5 are plotted in red, and genes with *P* < 0.05 and log_2_(-fold change null/WT) < −0.5 in blue. (**E, F**) Gene ontology (GO) cellular compartment analysis of the increased TE genes (red dots in C) and decreased TE genes (blue dots in C). (**G**) Percentage of Gln CAG codons in the coding sequence of each TE down gene (the blue dots in C) and TE up gene (the red dots in C) group. Data are presented as the mean ± s.e.m; n.s. = not significant (Mann–Whitney *U* test). (H, I) m^6^A (**H**) and m^6^Am (**I**) levels within poly(A)+ RNA. Total brain poly(A)+ RNA was digested to nucleosides using nuclease P1 and then subjected to LC–MS analysis. m^6^A and m^6^Am levels were normalized against uridine levels. Data are presented as the mean ± s.e.m. from *n* = 4 WT and *n* = 4 *Trmt10a* null 14-week-old male mice. *P< 0.05 (Welch's *t*-test). (**J**) Quantification of nascent cellular protein synthesis in WT and *TRMT10A* KO HEK293FT cells observed by ^35^S-methionine pulse-labeling. The original gel image is shown in Supplementary Figure S3C. Data represent the mean ± s.e.m. from a triplicate experiment. *P< 0.05 (Welch's *t*-test).

We conducted ribosome profiling experiments (36) to investigate the impact of tRNA m^1^G9 loss on translation (Figure 4A). To elucidate the effect of TRMT10A dysfunction on mRNA codon translation, we measured ribosome occupancy on each A-site codon. We observed increased ribosome occupancy on the glutamine CAG codon in the mutant brain (Figure 4B), indicating slowdown of ribosome traversal on this codon. This result was highly congruent with the reduction in tRNA^Gln(CUG)^ levels in the *Trmt10a* null brain (Figure 2A).

Next, we extended our analysis to the open reading frame (ORF). Here, we calculated translational efficiency (TE), which denotes the ribosome footprint number normalized against RNA abundance (as measured by mRNA-sequencing (RNA-seq)) (Figure 4A). We observed that TE decreased in 180 genes, and increased in 189 genes (Figure 4C, Supplementary Figure S4A), although changes in mRNA abundance were limited (Figure 4D).

We performed gene ontology (GO) analysis to better understand the gene groups associated with altered mRNA translation. From the perspective of cellular components, we observed enrichment of neuron-related GO terms such as ‘somatodendritic compartment’, ‘dendritic tree’, ‘neuron projection’, ‘postsynapse’, ‘synapse’ and ‘axon’ in the increasing TE gene group (189 red genes in Figure 4C) and in the decreasing TE gene group (180 blue genes in Figure 4C) (Figure 4E, F). By contrast, we found no GO terms related to mitochondria or the endoplasmic reticulum, emphasizing the importance of TRMT10A for translation of mRNAs related to neuronal structures. From the perspective of biological processes, cell signaling-related terms were enriched both in the increasing and decreasing TE gene groups (Supplementary Figure S4B).

Notably, when we compared the frequency of CAG codons between the decreasing and increasing TE gene groups, we did not observe a significant difference (Figure 4G); thus, the difference in TE between WT and null mice cannot be explained solely by the glutamine CAG codon content. Because TRMT10A affects not only tRNA m^1^G9 modification but also mRNA m^6^A and m^6^Am modifications (25), we measured the m^6^A and m^6^Am levels in poly(A)+ RNAs, which were selected using oligo(dT) beads (Figure 4A). Nucleoside analysis by mass spectrometry revealed a statistically non-significant increase in m^6^A, as well as a 40% increase in m^6^Am, in *Trmt10a* null brain poly(A) + RNAs (Figure 4H, I). Considering that the m^6^Am modification can promote translation (48), increased amounts of m^6^Am might be responsible, at least partially, for translational upregulation of the TE genes depicted in Figure 4C. In a previous study, *TRMT10A* knockdown increased overall translation (18). In line with this, our *TRMT10A* knockout cells showed increased levels of cellular translation, as shown by ^35^S-methionine labeling of nascent proteins (Figure 4J, Supplementary Figure S3C), a phenomenon that might be partly attributable to m^6^A-related mRNA modification(s).

Therefore, these results collectively demonstrate that TRMT10A is crucial for translation, especially of neuron-related mRNAs.

### TRMT10A dysfunction increases translation of the main coding sequence of *Atf4* mRNA, suggesting that reduced amounts of tRNA^iMet^ are available for translation

**Figure 5.**
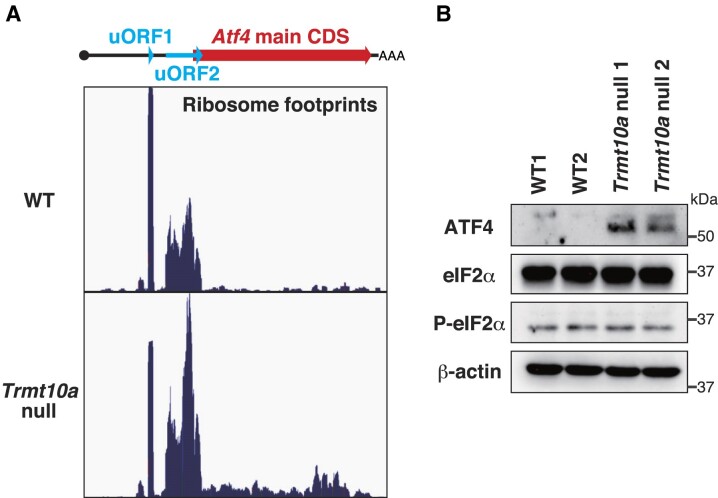
Reduced tRNA^iMet^ availability implicated by the release of *Atf4* main coding sequence translation in the *Trmt10a* null brain. (**A**) Ribosome footprints on *Atf4* mRNA. Reads from ribosome profiling of *n* = 3 WT and *n* = 3 *Trmt10a* null brains in Figure 4 were each added to obtain sufficient reads for visibility. Note that the translation of the *Atf4* main coding sequence (CDS) is suppressed in WT brains and released in *Trmt10a* null brains. (**B**) Western blot of ATF4, eIF2α, S51 phosphorylated eIF2α, and β-actin (loading control) in brains from *n* = 2 WT and *n* = 2 *Trmt10a* null 15-week-old males.

The reduction in the amount of tRNA^iMet^ (Figure 2A) led us to investigate the mechanism underlying translational regulation mediated by upstream (u)ORFs. In mice and humans, translation of the *Atf4* and *Atf5* (stress-responsive transcription factors) main coding sequence (CDS) is strongly suppressed by two uORFs (uORF1 and uORF2; see Figure 5A and Supplementary Figure S4C) (49–53). Under normal conditions, after translating uORF1, the ribosomal small subunit slides on the mRNA and quickly binds to the eIF2α-GTP-tRNA^iMet^ ternary complex to initiate translation of uORF2. Overlap of uORF2 with the main *Atf4* or *Atf5* CDS inhibits translation of the main CDS. By contrast, when availability of the eIF2α-GTP-tRNA^iMet^ complex becomes limited (e.g. due to eIF2α phosphorylation upon cellular stress), after translation of uORF1, the reduced concentration of the eIF2α-GTP-tRNA^iMet^ complex allows the ribosome subunit to bind to the eIF2α-GTP-tRNA^iMet^ complex after passing the uORF2 start codon and before reaching the main CDS start codon. This enables translation of the main CDS (49–53). Thus, we postulated that the reduced amount of tRNA^iMet^ in the *Trmt10a* null brain may phenocopy the translational response even in the absence of stress. Indeed, we observed an increase in ribosome footprints on the main CDS of *Atf4* in the *Trmt10a* null brain (Figure 5A, lower panel, Supplementary Figure S4D). Consistent with this, we observed accumulation of the ATF4 protein in *Trmt10a* null brains (Figure 5B). By contrast, although the *Atf5* CDS showed increasing trend toward an increased ribosomal footprint number (*P* = 0.066), there was no increase in ATF5 protein levels (Supplementary Figure S4A, S4C, S4D); this may be due to causes other than translation, including the balance between protein synthesis and degradation. Importantly, we observed neither a decrease in eIF2α protein levels nor an increase in eIF2α phosphorylation (Figure 5B). Therefore, TRMT10A deficiency can affect translation of the initiator methionine codon, at least with respect to translation of *Atf4* mRNA, which is strongly affected by availability of tRNA^iMet^.

### Impairment of TRMT10A causes brain dysfunction in mice

**Figure 6.**
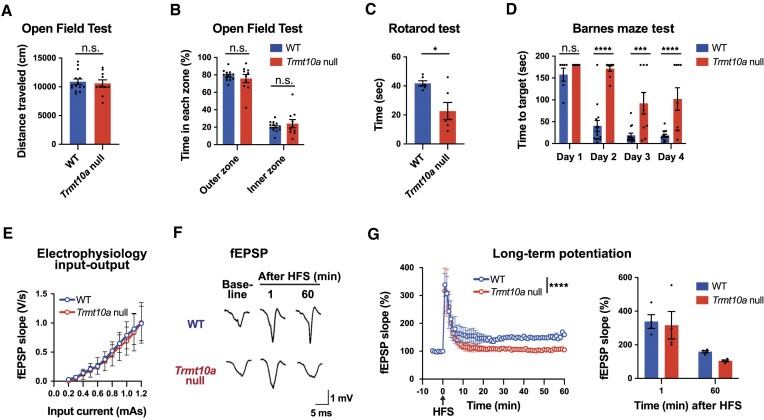
Impairment of neuronal functions in the *Trmt10a* null mice. (**A**) Distance traveled in the open-field test. Means ± s.e.m. from *n* = 14 WT and *n* = 10 *Trmt10a* null 12- to 15-week-old male mice. n.s. by Mann–Whitney test. (**B**) Time in the outer and inner zones in the open-field test. Means ± s.e.m. from *n* = 14 WT and *n* = 10 *Trmt10a* null 12- to 15-week-old males. n.s. by Mann–Whitney test. (**C**) Rotarod test. Means ± s.e.m. from *n* = 6 WT and *n* = 6 *Trmt10a* null 16- to 17-week-old males. *P < 0.05 by Mann–Whitney test. (**D**) Barnes maze test. Means ± s.e.m. from *n* = 14 WT and *n* = 9 *Trmt10a* null 14- to 17-week-old males. At day 1, although *n* = 14 WT and *n* = 9 *Trmt10a* null mice were used for the experiment, *n* = 6 WT and *n* = 7 *Trmt10a* null mice are shown because data were not initially acquired on day 1. ****P < 0.0001, ***P < 0.001 by two-way ANOVA followed by Sidak's test. (**E**) Input-output slope recorded in hippocampal slices. Means ± s.e.m. from *n* = 4 WT and *n* = 4 *Trmt10a* null 16- to 22-week-old males. (**F**) Representative traces of field excitatory postsynaptic potentials (fEPSPs) detected before and after the presentation of high-frequency stimuli (HFS). HFS was administered at 0 min to induce long-term potentiation (LTP). (**G**) Left, HFS-evoked LTP in hippocampal slices. Right, fEPSP slope quantification at 1 min and 60 min after HFS administration. Data represent the mean ± s.e.m. (derived from the same mice as in Figure 6E). ****P < 0.0001 (two-way ANOVA).

Given the aberrant translation in the *Trmt10a* null brain (Figures 4 and 5), as well as the smaller postsynaptic densities in the hippocampus (Figure 3J, K), we next investigated whether impairment of TRMT10A causes brain dysfunction. First, we subjected mice to the open-field test and found that spontaneous locomotor activity and fear-like behaviors were not affected (Figure 6A, B). However, the rotarod test revealed that *Trmt10a* null mice could not continue walking and staying on the rotating rod (Figure 6C), suggestive of reduced motor coordination by the central nervous system. We then performed the Barnes maze test, and observed that *Trmt10a* null mice had difficulty learning and memorizing the location of the hiding box (Figure 6D), suggesting reduced spatial learning ability. Because hippocampal long-term potentiation (LTP) is critical for learning and memory, we next performed electrophysiological experiments to assess neuronal plasticity. We found no changes in the input-output slope curve in the field excitatory postsynaptic potential (fEPSP), suggesting similar basic electrophysiological properties across both WT and *Trmt10a* null hippocampal synapses (Figure 6E and ‘baseline’ in Figure 6F). We then administered high frequency stimuli (HFS) at 0 min to induce LTP in hippocampal slices. There was no difference in the maximum fEPSP slope of the WT and *Trmt10a* null hippocampal slices after administering the HFS (1 min after HFS; Figure 6G). In the WT hippocampus, the fEPSP slope was maintained at >150%, at least until 60 min post-administration of the HFS, suggesting successful induction of LTP; however, maintenance of LTP was disrupted in the *Trmt10a* null hippocampus at 60 min post-administration of HFS (Figure 6G), suggesting impaired hippocampal neuronal plasticity in *Trmt10a* null mice.

Taken together, these data demonstrate that brain functions are impaired in *Trmt10a* null mice.

## Discussion

Among the >50 tRNA modification enzymes in which mutations are associated with human disease, mutations in >20 of them are associated with brain-related diseases. Despite recent efforts, we still do not know exactly why the brain is most affected, or how loss of tRNA modifications impacts the brain. Recently, we reported that upon loss of mouse FTSJ1, a 2′-*O*-methyltransferase for tRNA positions 32 and 34, tRNA^Phe^ was degraded only in the brain and not in other tissues such as the kidney and liver; moreover, we observed slowdown of Phe codon translation in the brain (8). Thus, in the case of FTSJ1-mediated tRNA methylation, a brain-preferential phenotype upon FTSJ1 loss is thought to be at least partly due to brain-specific tRNA^Phe^ degradation. By contrast, our current study demonstrated that in the case of TRMT10A, loss of TRMT10A universally reduced the levels of tRNA^iMet^ and tRNA^Gln(CUG)^ in various tissues and cells. Because body size was smaller in the *Trmt10a* null mice than in WT mice, the physiological phenotype was not brain-specific; nevertheless, at the tissue level, the strongest phenotype was observed in the brain. Therefore, the brain preference of disorders upon loss of a tRNA modification enzyme persists, regardless of differences in tRNA modification enzymes. Furthermore, both the *Trmt10a* null mice and *Ftsj1* KO mice (8) showed smaller postsynaptic densities and impaired synaptic plasticity in the hippocampus, which explained the memory impairment displayed by the mice.

The brain preference of a disease phenotype in genetic diseases is not limited to the loss of tRNA modification. In fact, it is widely seen in mutations of various translation-related factors, such as aminoacyl-tRNA synthetases, translational initiation factors, and translational elongation factors (54,55). Local translation is a neuron-specific event. In axon terminals and dendrites close to synapses, the local translation level increases several fold upon transmission of strong stimuli (56), and is essential for synaptic plasticity (57). Future studies on the effect of tRNA modification loss in neuronal local translations near the synapses will be vital to gaining further insight into the brain preference of tRNA modopathies.

uORFs are present in approximately half of human and mouse transcripts and repress or promote mRNA translation of thousands of genes, depending on the uORF number and structure (e.g. distance and overlap with the main CDS) (49,58). Translational regulation of *Atf4* and *Atf5* mRNAs by uORFs is conserved between human and mouse, and translation of the main CDS of these two mRNAs are strongly repressed in normal states and released upon reductions in the concentration of the tRNA^iMet^-eIF2α-GTP ternary complex (49,51–53). Our present study showed a decrease in the tRNA^iMet^ level to approximately 50% in the *Trmt10a* null brain, which likely released *Atf4* translation in the *Trmt10a* null brain. Recent technologies have enabled the discovery that mRNAs involved in synapse organization and axon development are highly enriched for uORF translation in certain neurons (59). In addition to abundant and strongly regulated mRNAs such as *Atf4*, improved technologies may reveal sensitive differences in the translation of various neuronal mRNAs upon loss of a tRNA modification.

Originally, we conducted our TRMT10A experiment to examine the function of tRNA modification in the brain. However, during the progress of our study, TRMT10A was reported to also be involved in FTO-mediated mRNA m^6^A and m^6^Am demethylation (25). In line with this report, we confirmed the effect of TRMT10A on mRNA m^6^Am (Figure 4I). Our study revealed that TRMT10A has a larger impact on translation than on mRNA levels (Figure 4C, D). Considering that m^6^A-modified RNAs accumulate in the synaptic compartment following learning (60), further studies from the perspective of mRNA m^6^A modification would deepen our understanding of how TRMT10A deficiency causes brain disorders.

Our data suggest that in the *Trmt10a* null brains, ribosomes are slowing down on glutamine (CAG) codons, which may be explained by the reduction in the tRNA^Gln(CUG)^ level. However, when we compared the frequency of CAG codons between the decreasing and increasing TE genes, we did not observe a significant difference (Figure 4G). In addition, in a previous study, *TRMT10A* knockdown resulted in increased overall translation (18). In line with this report, *TRMT10A* knockout cells showed increased cellular translation, as shown by ^35^S-methionine labeling of nascent proteins (Figure 4J, Supplementary Figure S3C), which might be attributable, at least in part, to increased m^6^Am or m^6^A modifications. Additionally, a previous study showed that patient-derived *TRMT10A*-deficient lymphoblasts contained a tRNA^Gln^ 5′ fragment (14); however, using a probe that detects essentially the same region of the tRNA^Gln^ 5′ fragment as the previous study (14), we did not observe an increase in the amount of tRNA^Gln^ 5′ fragment in the *Trmt10a* null brain (Supplementary Figure S2B, S2D). Nevertheless, this does not deny the possible presence of fragments from other tRNAs; this will be a subject for future studies. Taken together, the translational changes due to TRMT10A dysfunction cannot be explained by glutamine CAG codon frequency only; rather, they may derive from the combined effects of CAG codon frequency-derived perturbation of translational elongation, m^6^A/m^6^Am-mediated translational upregulation, the possible contribution of tRNA fragments, and tRNA^iMet^-mediated perturbation of translational initiation.

Similar to patients with *TRMT10A* mutations, *Trmt10a* null mice showed smaller body size (Figure 1E–G). To find the cause of this phenomenon, we measured GH, IGF-1, and TSH levels (GH and TSH are secreted by the pituitary gland), but neither showed a clear difference between *Trmt10a* null and WT mice (Supplementary Figure S1A). Thus, to better understand the cause of smaller body size in *Trmt10a* null mice, we may need to investigate translational perturbation within the whole body. We noted that levels of adrenocorticotrophic hormone (ACTH), a pituitary gland-derived hormone not directly related to growth, were lower in *Trmt10a* null mice than in WT mice (Supplementary Figure S1B); therefore, we cannot completely rule out the possibility of a partial decline in the function of the pituitary gland, which may affect the body in multiple ways. In addition to *TRMT10A*, mutations in various tRNA modification enzyme genes (*TRMT5*, *NSUN2*, *NSUN3*, *WDR4*, *PUS3, PUS7*, *CTU2*, *YRDC*, *OSGEP*, *TP53RK*, *TPRKB*, *LAGE3*, *MTO1*, *GTPBP3*, *THG1L*, and *TRIT1*) cause short stature (5), and all of these mutations are associated with brain disorders. Thus, identifying whether defects of these tRNA modification enzymes cause short stature via perturbation of translation throughout the whole body, or via aberration of hormone level(s), may be an important subject for future studies.

Despite the presence of m^1^G9 in approximately 40% of the various cytoplasmic tRNA species, the mechanism underlying how TRMT10A dysfunction specifically reduces tRNA^iMet^ and tRNA^Gln(CUG)^ remains unclear. In humans and mice, the base at tRNA position nine (N9) is almost always a purine (Supplementary Table S1) (11,13), which in some cases may form a N9-N23-N12 base triplet (61,62); thus, studies involving direct structural analysis or structural probing experiments are needed to assess whether the m^1^G9 modification affects the base triplet structure and overall structure of tRNA. tRNAs with aberrant structures can be degraded by pathways such as 1) the TRF4/RRP6-dependent nuclear surveillance pathway via polyadenylation and degradation by the nuclear exosome (63), 2) the rapid tRNA decay pathway (64), or 3) via endonucleolytic cleavage by the ribonuclease A family (65). In our tRNA-seq and tRNA northern blots, we did not observe a detectable amount of poly(A) tail, repeating CCACCA tail (66), or an increase in 5′tRNA^Gln^ fragments, in RNA from *Trmt10a* null tissue (Figure 2A, H, Supplementary Figure S2D). Although we observed slightly increased traces of potential 5′ exonucleolytic degradation in the *Trmt10a* null brain (Figure 2H) (14), further studies are required to determine which pathway is responsible for the specific reduction in tRNA^iMet^ and tRNA^Gln(CUG)^ in *Trmt10a* null tissues and cells.

In summary, we generated *Trmt10a* null mice and demonstrated that TRMT10A dysfunction induces a universal reduction in tRNA^iMet^ and tRNA^Gln(CUG)^ levels, which is associated with translational aberrations, smaller postsynaptic densities, impaired synaptic plasticity, and impaired brain function. Our findings deepen our understanding of how loss of tRNA modification affects translation and causes brain impairment.

## Supplementary Material

gkae520_Supplemental_File

## Data Availability

All data and cell resources presented in this study are available upon reasonable request. Sequencing data were submitted to Sequence Read Archive (SRA) under accession number PRJNA1034671 (https://www.ncbi.nlm.nih.gov/sra/PRJNA1034671).

## References

[1] Crick F. On protein synthesis. Symp. Soc. Exp. Biol. 1958; 12:138–163. 13580867

[2] Hoagland M.B. , Stephenson M.L., Scott J.F., Hecht L.I., Zamecnik P.C. A soluble ribonucleic acid intermediate in protein synthesis. J. Biol. Chem. 1958; 231:241–257. 13538965

[3] Boccaletto P. , Stefaniak F., Ray A., Cappannini A., Mukherjee S., Purta E., Kurkowska M., Shirvanizadeh N., Destefanis E., Groza P. et al. MODOMICS: a database of RNA modification pathways. 2021 update. Nucleic Acids Res. 2022; 50:D231–D235. 34893873 10.1093/nar/gkab1083 PMC8728126

[4] Suzuki T. The expanding world of tRNA modifications and their disease relevance. Nat. Rev. Mol. Cell Biol. 2021; 22:375–392. 33658722 10.1038/s41580-021-00342-0

[5] Chujo T. , Tomizawa K. Human transfer RNA modopathies: diseases caused by aberrations in transfer RNA modifications. FEBS J. 2021; 288:7096–7122. 33513290 10.1111/febs.15736 PMC9255597

[6] de Crecy-Lagard V. , Boccaletto P., Mangleburg C.G., Sharma P., Lowe T.M., Leidel S.A., Bujnicki J.M. Matching tRNA modifications in humans to their known and predicted enzymes. Nucleic Acids Res. 2019; 47:2143–2159. 30698754 10.1093/nar/gkz011 PMC6412123

[7] Delaunay S. , Helm M., Frye M. RNA modifications in physiology and disease: towards clinical applications. Nat. Rev. Genet. 2024; 25:104–122. 37714958 10.1038/s41576-023-00645-2

[8] Nagayoshi Y. , Chujo T., Hirata S., Nakatsuka H., Chen C.W., Takakura M., Miyauchi K., Ikeuchi Y., Carlyle B.C., Kitchen R.R. et al. Loss of Ftsj1 perturbs codon-specific translation efficiency in the brain and is associated with X-linked intellectual disability. Sci. Adv. 2021; 7:eabf3072. 33771871 10.1126/sciadv.abf3072 PMC7997516

[9] Freude K. , Hoffmann K., Jensen L.R., Delatycki M.B., des Portes V., Moser B., Hamel B., van Bokhoven H., Moraine C., Fryns J.P. et al. Mutations in the FTSJ1 gene coding for a novel S-adenosylmethionine-binding protein cause nonsyndromic X-linked mental retardation. Am. J. Hum. Genet. 2004; 75:305–309. 15162322 10.1086/422507 PMC1216064

[10] Guy M.P. , Shaw M., Weiner C.L., Hobson L., Stark Z., Rose K., Kalscheuer V.M., Gecz J., Phizicky E.M. Defects in tRNA anticodon loop 2’-O-methylation are implicated in nonsyndromic X-linked intellectual dsability due to mutations in FTSJ1. Hum. Mutat. 2015; 36:1176–1187. 26310293 10.1002/humu.22897 PMC4643400

[11] Chan P.P. , Lowe T.M. GtRNAdb 2.0: an expanded database of transfer RNA genes identified in complete and draft genomes. Nucleic Acids Res. 2016; 44:D184–D189. 26673694 10.1093/nar/gkv1309 PMC4702915

[12] Clark W.C. , Evans M.E., Dominissini D., Zheng G., Pan T. tRNA base methylation identification and quantification via high-throughput sequencing. RNA. 2016; 22:1771–1784. 27613580 10.1261/rna.056531.116 PMC5066629

[13] Westhof E. , Thornlow B., Chan P.P., Lowe T.M. Eukaryotic tRNA sequences present conserved and amino acid-specific structural signatures. Nucleic Acids Res. 2022; 50:4100–4112. 35380696 10.1093/nar/gkac222 PMC9023262

[14] Cosentino C. , Toivonen S., Diaz Villamil E., Atta M., Ravanat J.L., Demine S., Schiavo A.A., Pachera N., Deglasse J.P., Jonas J.C. et al. Pancreatic beta-cell tRNA hypomethylation and fragmentation link TRMT10A deficiency with diabetes. Nucleic Acids Res. 2018; 46:10302–10318. 30247717 10.1093/nar/gky839 PMC6212784

[15] Vilardo E. , Amman F., Toth U., Kotter A., Helm M., Rossmanith W. Functional characterization of the human tRNA methyltransferases TRMT10A and TRMT10B. Nucleic Acids Res. 2020; 48:6157–6169. 32392304 10.1093/nar/gkaa353 PMC7293042

[16] Brener A. , Zeitlin L., Wilnai Y., Birk O.S., Rosenfeld T., Chorna E., Lebenthal Y. Looking for the skeleton in the closet-rare genetic diagnoses in patients with diabetes and skeletal manifestations. Acta Diabetol. 2022; 59:711–719. 35137278 10.1007/s00592-022-01854-7

[17] Gillis D. , Krishnamohan A., Yaacov B., Shaag A., Jackman J.E., Elpeleg O. TRMT10A dysfunction is associated with abnormalities in glucose homeostasis, short stature and microcephaly. J. Med. Genet. 2014; 51:581–586. 25053765 10.1136/jmedgenet-2014-102282

[18] Igoillo-Esteve M. , Genin A., Lambert N., Desir J., Pirson I., Abdulkarim B., Simonis N., Drielsma A., Marselli L., Marchetti P. et al. tRNA methyltransferase homolog gene TRMT10A mutation in young onset diabetes and primary microcephaly in humans. PLos Genet. 2013; 9:e1003888. 24204302 10.1371/journal.pgen.1003888 PMC3814312

[19] Lin H. , Zhou X., Chen X., Huang K., Wu W., Fu J., Li Y., Polychronakos C., Dong G.P. tRNA methyltransferase 10 homologue A (TRMT10A) mutation in a Chinese patient with diabetes, insulin resistance, intellectual deficiency and microcephaly. BMJ Open Diabetes Res Care. 2020; 8:e001601. 10.1136/bmjdrc-2020-001601 PMC7569974 33067246

[20] Narayanan M. , Ramsey K., Grebe T., Schrauwen I., Szelinger S., Huentelman M., Craig D., Narayanan V., Group C.R.R. Case Report: compound heterozygous nonsense mutations in TRMT10A are associated with microcephaly, delayed development, and periventricular white matter hyperintensities. F1000Res. 2015; 4:912. 26535115 10.12688/f1000research.7106.1 PMC4617320

[21] Siklar Z. , Kontbay T., Colclough K., Patel K.A., Berberoglu M. Expanding the Phenotype of TRMT10A Mutations: case Report and a Review of the Existing Cases. J. Clin. Res. Pediatr. Endocrinol. 2023; 15:90–96. 34541035 10.4274/jcrpe.galenos.2021.2021.0110 PMC9976169

[22] Stern E. , Vivante A., Barel O., Levy-Shraga Y. TRMT10A mutation in a child with diabetes, short stature, microcephaly and hypoplastic kidneys. J. Clin. Res. Pediatr. Endocrinol. 2022; 14:227–232. 33448213 10.4274/jcrpe.galenos.2020.2020.0265 PMC9176091

[23] Yew T.W. , McCreight L., Colclough K., Ellard S., Pearson E.R. tRNA methyltransferase homologue gene TRMT10A mutation in young adult-onset diabetes with intellectual disability, microcephaly and epilepsy. Diabet. Med. 2016; 33:e21–e25. 26526202 10.1111/dme.13024 PMC4995728

[24] Zung A. , Kori M., Burundukov E., Ben-Yosef T., Tatoor Y., Granot E. Homozygous deletion of TRMT10A as part of a contiguous gene deletion in a syndrome of failure to thrive, delayed puberty, intellectual disability and diabetes mellitus. Am. J. Med. Genet. A. 2015; 167A:3167–3173. 26297882 10.1002/ajmg.a.37341

[25] Ontiveros R.J. , Shen H., Stoute J., Yanas A., Cui Y., Zhang Y., Liu K.F. Coordination of mRNA and tRNA methylations by TRMT10A. Proc. Natl. Acad. Sci. USA. 2020; 117:7782–7791. 32213595 10.1073/pnas.1913448117 PMC7149399

[26] Jiang X. , Liu B., Nie Z., Duan L., Xiong Q., Jin Z., Yang C., Chen Y. The role of m6A modification in the biological functions and diseases. Signal Transduct. Target. Ther. 2021; 6:74. 33611339 10.1038/s41392-020-00450-x PMC7897327

[27] Wei J. , Liu F., Lu Z., Fei Q., Ai Y., He P.C., Shi H., Cui X., Su R., Klungland A. et al. Differential m(6)A, m(6)A(m), and m(1)A demethylation mediated by FTO in the cell nucleus and cytoplasm. Mol. Cell. 2018; 71:973–985. 30197295 10.1016/j.molcel.2018.08.011 PMC6151148

[28] Fukuda H. , Chujo T., Wei F.Y., Shi S.L., Hirayama M., Kaitsuka T., Yamamoto T., Oshiumi H., Tomizawa K. Cooperative methylation of human tRNA3Lys at positions A58 and U54 drives the early and late steps of HIV-1 replication. Nucleic Acids Res. 2021; 49:11855–11867. 34642752 10.1093/nar/gkab879 PMC8599865

[29] Shalem S. , Sanjana N.E., Hartenian E., Shi X., Scott D.A., Mikkelsen T.S., Heckl D., Ebert B.L., Root D.E., Doench J.G. et al. Genome-scale CRISPR-Cas9 knockout screening in human cells. Science. 2014; 343:84–87. 24336571 10.1126/science.1247005 PMC4089965

[30] Murakami Y. , Wei F.Y., Kawamura Y., Horiguchi H., Kadomatsu T., Miyata K., Miura K., Oike Y., Ando Y., Ueda M. et al. NSUN3-mediated mitochondrial tRNA 5-formylcytidine modification is essential for embryonic development and respiratory complexes in mice. Commun. Biol. 2023; 6:307. 36949224 10.1038/s42003-023-04680-x PMC10033821

[31] Nagayoshi Y. , Nishiguchi K., Yamamura R., Chujo T., Oshiumi H., Nagata H., Kaneko H., Yamamoto K., Nakata H., Sakakida K. et al. t(6)A and ms(2)t(6)A modified nucleosides in serum and urine as strong candidate biomarkers of COVID-19 infection and severity. Biomolecules. 2022; 12:1233. 36139072 10.3390/biom12091233 PMC9496545

[32] Chen C.W. , Tanaka M. Genome-wide translation profiling by ribosome-bound tRNA capture. Cell Rep. 2018; 23:608–621. 29642016 10.1016/j.celrep.2018.03.035

[33] Galaxy C. The Galaxy platform for accessible, reproducible and collaborative biomedical analyses: 2022 update. Nucleic Acids Res. 2022; 50:W345–W351. 35446428 10.1093/nar/gkac247 PMC9252830

[34] Ahmad R.N.R. , Zhang L.T., Morita R., Tani H., Wu Y., Chujo T., Ogawa A., Harada R., Shigeta Y., Tomizawa K. et al. Pathological mutations promote proteolysis of mitochondrial tRNA-specific 2-thiouridylase 1 (MTU1) via mitochondrial caseinolytic peptidase (CLPP). Nucleic Acids Res. 2024; 52:1341–1358. 38113276 10.1093/nar/gkad1197 PMC10853782

[35] Janssen B.D. , Diner E.J., Hayes C.S. Analysis of aminoacyl- and peptidyl-tRNAs by gel electrophoresis. Methods Mol. Biol. 2012; 905:291–309. 22736012 10.1007/978-1-61779-949-5_19 PMC3682404

[36] Ingolia N.T. , Ghaemmaghami S., Newman J.R., Weissman J.S. Genome-wide analysis in vivo of translation with nucleotide resolution using ribosome profiling. Science. 2009; 324:218–223. 19213877 10.1126/science.1168978 PMC2746483

[37] Subramanian A. , Tamayo P., Mootha V.K., Mukherjee S., Ebert B.L., Gillette M.A., Paulovich A., Pomeroy S.L., Golub T.R., Lander E.S. et al. Gene set enrichment analysis: a knowledge-based approach for interpreting genome-wide expression profiles. Proc. Natl. Acad. Sci. U.S.A. 2005; 102:15545–15550. 16199517 10.1073/pnas.0506580102 PMC1239896

[38] Robinson J.T. , Thorvaldsdottir H., Winckler W., Guttman M., Lander E.S., Getz G., Mesirov J.P. Integrative genomics viewer. Nat. Biotechnol. 2011; 29:24–26. 21221095 10.1038/nbt.1754 PMC3346182

[39] Takesue Y. , Wei F.Y., Fukuda H., Tanoue Y., Yamamoto T., Chujo T., Shinojima N., Yano S., Morioka M., Mukasa A. et al. Regulation of growth hormone biosynthesis by Cdk5 regulatory subunit associated protein 1-like 1 (CDKAL1) in pituitary adenomas. Endocr. J. 2019; 66:807–816. 31189758 10.1507/endocrj.EJ18-0536

[40] Yakita M. , Chujo T., Wei F.Y., Hirayama M., Kato K., Takahashi N., Naganuma K., Nagata M., Kawahara K., Nakayama H. et al. Extracellular N6-isopentenyladenosine (i6A) addition induces cotranscriptional i6A incorporation into ribosomal RNAs. RNA. 2022; 28:1013–1027. 35414588 10.1261/rna.079176.122 PMC9202588

[41] Wei F.Y. , Suzuki T., Watanabe S., Kimura S., Kaitsuka T., Fujimura A., Matsui H., Atta M., Michiue H., Fontecave M. et al. Deficit of tRNA(Lys) modification by Cdkal1 causes the development of type 2 diabetes in mice. J. Clin. Invest. 2011; 121:3598–3608. 21841312 10.1172/JCI58056 PMC3163968

[42] Franklin K.B. , Paxinos G. Paxinos and Franklin's The mouse brain in stereotaxic coordinates. 2013; 4th edn. Academic Press.

[43] Fukuda T. , Aika Y., Heizmann C.W., Kosaka T. GABAergic axon terminals at perisomatic and dendritic inhibitory sites show different immunoreactivities against two GAD isoforms, GAD67 and GAD65, in the mouse hippocampus: a digitized quantitative analysis. J. Comp. Neurol. 2008; 395:177–194. 10.1002/(sici)1096-9861(19980601)395:2<177::aid-cne3>3.0.co;2-# 9603371

[44] Shigematsu N. , Yamamoto K., Higuchi S., Fukuda T. An immunohistochemical study on a unique colocalization relationship between substance P and GABA in the central nucleus of amygdala. Brain Res. 2008; 1198:55–67. 18243164 10.1016/j.brainres.2007.12.064

[45] Pitts M.W. Barnes Maze Procedure for Spatial Learning and Memory in Mice. Bio. Protoc. 2018; 8:e2744. 10.21769/BioProtoc.2744 PMC5891830 29651452

[46] Deacon R.M. Measuring motor coordination in mice. J. Vis. Exp. 2013; 75:e2609. 10.3791/2609 PMC3724562 23748408

[47] Asamitsu S. , Yabuki Y., Ikenoshita S., Kawakubo K., Kawasaki M., Usuki S., Nakayama Y., Adachi K., Kugoh H., Ishii K. et al. CGG repeat RNA G-quadruplexes interact with FMRpolyG to cause neuronal dysfunction in fragile X-related tremor/ataxia syndrome. Sci. Adv. 2021; 7:eabd9440. 33523882 10.1126/sciadv.abd9440 PMC7806243

[48] Akichika S. , Hirano S., Shichino Y., Suzuki T., Nishimasu H., Ishitani R., Sugita A., Hirose Y., Iwasaki S., Nureki O. et al. Cap-specific terminal N (6)-methylation of RNA by an RNA polymerase II-associated methyltransferase. Science. 2019; 363:eaav0080. 30467178 10.1126/science.aav0080

[49] Dever T.E. , Ivanov I.P., Hinnebusch A.G. Translational regulation by uORFs and start codon selection stringency. Genes Dev. 2023; 37:474–489. 37433636 10.1101/gad.350752.123 PMC10393191

[50] Kashiwagi K. , Shichino Y., Osaki T., Sakamoto A., Nishimoto M., Takahashi M., Mito M., Weber F., Ikeuchi Y., Iwasaki S. et al. eIF2B-capturing viral protein NSs suppresses the integrated stress response. Nat. Commun. 2021; 12:7102. 34876589 10.1038/s41467-021-27337-x PMC8651795

[51] Lu P.D. , Harding H.P., Ron D. Translation reinitiation at alternative open reading frames regulates gene expression in an integrated stress response. J. Cell Biol. 2004; 167:27–33. 15479734 10.1083/jcb.200408003 PMC2172506

[52] Vattem K.M. , Wek R.C. Reinitiation involving upstream ORFs regulates ATF4 mRNA translation in mammalian cells. Proc. Natl. Acad. Sci. U.S.A. 2004; 101:11269–11274. 15277680 10.1073/pnas.0400541101 PMC509193

[53] Zhou D. , Palam L.R., Jiang L., Narasimhan J., Staschke K.A., Wek R.C. Phosphorylation of eIF2 directs ATF5 translational control in response to diverse stress conditions. J. Biol. Chem. 2008; 283:7064–7073. 18195013 10.1074/jbc.M708530200

[54] Kapur M. , Ackerman S.L. mRNA translation gone Awry: translation fidelity and neurological disease. Trends Genet. 2018; 34:218–231. 29352613 10.1016/j.tig.2017.12.007 PMC5834357

[55] Kapur M. , Monaghan C.E., Ackerman S.L. Regulation of mRNA translation in neurons—a matter of life and death. Neuron. 2017; 96:616–637. 29096076 10.1016/j.neuron.2017.09.057 PMC5693308

[56] Sun C. , Nold A., Fusco C.M., Rangaraju V., Tchumatchenko T., Heilemann M., Schuman E.M. The prevalence and specificity of local protein synthesis during neuronal synaptic plasticity. Sci. Adv. 2021; 7:eabj0790. 34533986 10.1126/sciadv.abj0790 PMC8448450

[57] Rangaraju V. , Tom Dieck S., Schuman E.M. Local translation in neuronal compartments: how local is local?. EMBO Rep. 2017; 18:693–711. 28404606 10.15252/embr.201744045 PMC5412868

[58] Calvo S.E. , Pagliarini D.J., Mootha V.K. Upstream open reading frames cause widespread reduction of protein expression and are polymorphic among humans. Proc. Natl. Acad. Sci. U.S.A. 2009; 106:7507–7512. 19372376 10.1073/pnas.0810916106 PMC2669787

[59] Froberg J.E. , Durak O., Macklis J.D. Development of nanoRibo-seq enables study of regulated translation by cortical neuron subtypes, showing uORF translation in synaptic-axonal genes. Cell Rep. 2023; 42:112995. 37624698 10.1016/j.celrep.2023.112995 PMC10591829

[60] Madugalle S.U. , Liau W.S., Zhao Q., Li X., Gong H., Marshall P.R., Periyakaruppiah A., Zajaczkowski E.L., Leighton L.J., Ren H. et al. Synapse-enriched m(6)A-modified Malat1 interacts with the novel m(6)A reader, DPYSL2, and is required for fear-extinction memory. J. Neurosci. 2023; 43:7084–7100. 37669863 10.1523/JNEUROSCI.0943-23.2023 PMC10601377

[61] Kim S.H. , Suddath F.L., McPherson A., Sussman J.L., Wang A.H.J., Seeman N.C., Rich A. Three-dimensional tertiary structure of yeast phenylalanine transfer RNA. Science. 1974; 185:435–440. 4601792 10.1126/science.185.4149.435

[62] Oliva R. , Cavallo L., Tramontano A. Accurate energies of hydrogen bonded nucleic acid base pairs and triplets in tRNA tertiary interactions. Nucleic Acids Res. 2006; 34:865–879. 16461956 10.1093/nar/gkj491 PMC1361619

[63] Kadaba S. , Krueger A., Trice T., Krecic A.M., Hinnebusch A.G., Anderson J. Nuclear surveillance and degradation of hypomodified initiator tRNAMet in S. cerevisiae. Genes Dev. 2004; 18:1227–1240. 15145828 10.1101/gad.1183804 PMC420349

[64] Alexandrov A. , Chernyakov I., Gu W., Hiley S.L., Hughes T.R., Grayhack E.J., Phizicky E.M. Rapid tRNA decay can result from lack of nonessential modifications. Mol. Cell. 2006; 21:87–96. 16387656 10.1016/j.molcel.2005.10.036

[65] Blanco S. , Dietmann S., Flores J.V., Hussain S., Kutter C., Humphreys P., Lukk M., Lombard P., Treps L., Popis M. et al. Aberrant methylation of tRNAs links cellular stress to neuro-developmental disorders. EMBO J. 2014; 33:2020–2039. 25063673 10.15252/embj.201489282 PMC4195770

[66] Wilusz J.E. , Whipple J.M., Phisicky E.M., Sharp P.A. tRNAs marked with CCACCA are targeted for degradation. Science. 2011; 334:817–820. 22076379 10.1126/science.1213671 PMC3273417

[67] Gillum A.M. , Roe B.A., Anandaraj J.S., RajBhandary U.L. Nucleotide sequence of human placenta cytoplasmic initiator tRNA. Cell. 1975; 6:407–413. 1052774 10.1016/0092-8674(75)90190-7

[68] Piper P.W. , Clark F.C. The nucleotide sequence of the cytoplasmic initiator transfer RNA of a mouse myeloma cell. Eur. J. Biochem. 1974; 45:589–600. 4369331 10.1111/j.1432-1033.1974.tb03585.x

