# Supplementary material for: TRMT10A dysfunction perturbs codon translation of initiator methionine and glutamine and impairs brain functions in mice
Source: Nucleic Acids Res. 2024 Jul 2;52(15):9230–46. doi: 10.1093/nar/gkae520 (PMC11347157; doi:10.1093/nar/gkae520)
Supplement: gkae520_Supplemental_File [file gkae520_supplemental_file.pdf]

Supplementary Data for

TRMT10A dysfunction perturbs codon translation of initiator methionine and glutamine and impairs brain functions in mice

Roland Tresky, Yuta Miyamoto, Yu Nagayoshi, Yasushi Yabuki, Kimi Araki, Yukie Takahashi, Yoshihiro Komohara, Huicong Ge, Kayo Nishiguchi, Takaichi Fukuda, Hitomi Kaneko, Nobuko Maeda, Jin Matsuura, Shintaro Iwasaki, Kourin Sakakida, Norifumi Shioda, Fan-Yan Wei, Kazuhito Tomizawa* and Takeshi Chujo*

*Corresponding authors.
Email: tchujo@kumamoto-u.ac.jp (T.C.), tomikt@kumamoto-u.ac.jp (K.T.)

This file includes:

Supplementary Figures 1 to 4.

Supplementary Tables 1 to 4.

Supplementary Figure 1. Hormone levels in *Trmt10a* null and WT mice

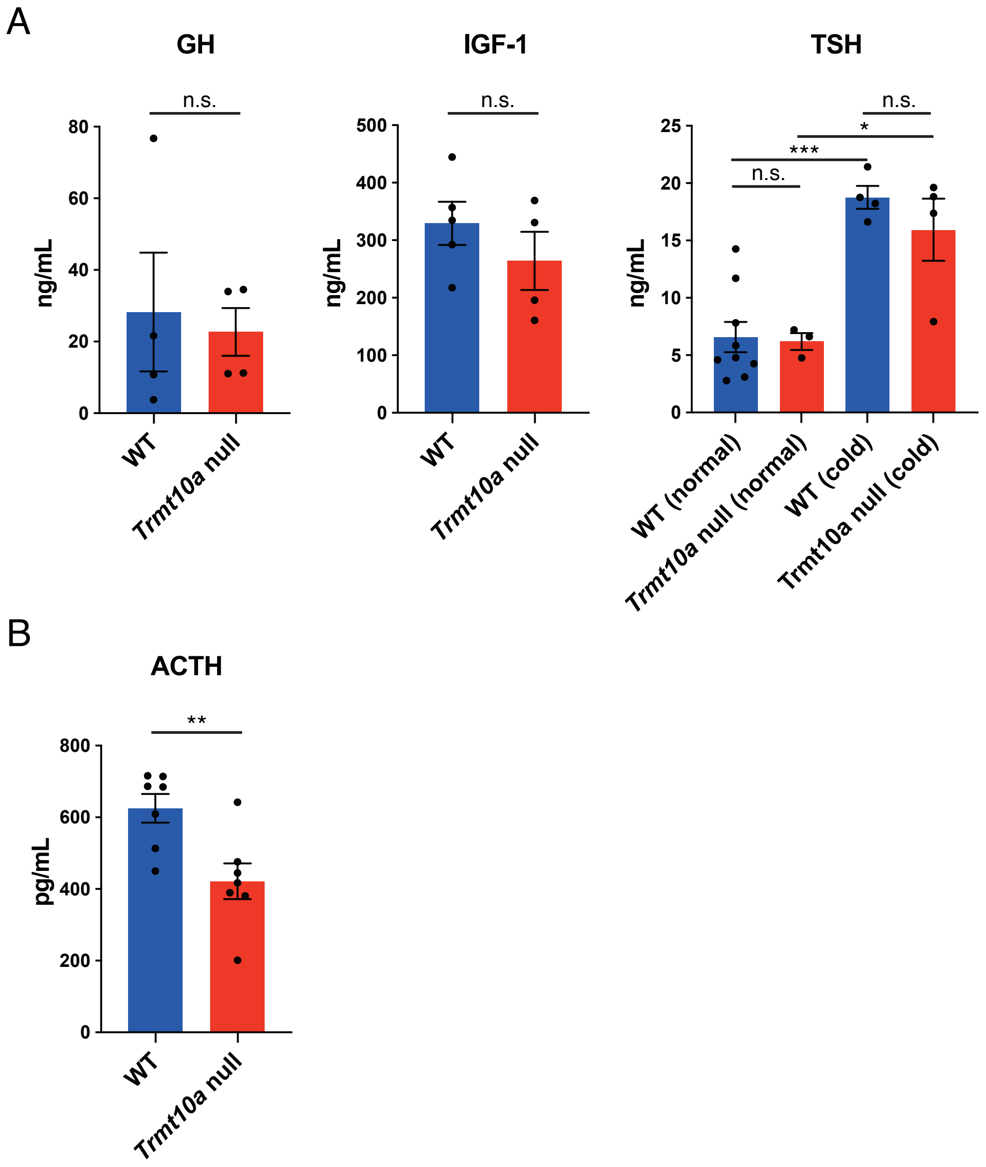

Supplementary Figure 1. Hormone levels in *Trmt10a* null and WT mice.
(A) Growth-related hormone levels. GH levels were measured in serum samples from $n = 4$ WT and $n = 4$ *Trmt10a* null 4-week-old mice. IGF-1 levels were measured in serum samples from $n = 5$ WT and $n = 4$ *Trmt10a* null 7–10-week-old mice. TSH levels were measured under normal conditions, or after cold treatment (mice placed in 4˚C chamber for 30 min) that elicits acute release of TSH. Serum was collected from 7–10-week-old WT mice ($n = 9$) under normal conditions, from *Trmt10a* null mice under normal conditions ($n = 3$), from WT mice ($n = 4$) after cold shock, and from *Trmt10a* null mice ($n = 4$) after cold treatment. **(B)** ACTH hormone levels were measured in serum samples from $n = 7$ WT and $n = 7$ *Trmt10a* null 10-week-old mice. For GH, IGF-1, and ACTH, $^{**}P < 0.01$ or not significant by Welch's *t*-test. For TSH, $^{***}P < 0.001$, $^{*}P < 0.05$, or not significant by 2-way ANOVA followed by Sidak's multiple comparison test.

Supplementary Figure 2. Northern blot analysis of *Trmt10a* null and WT mouse brains.

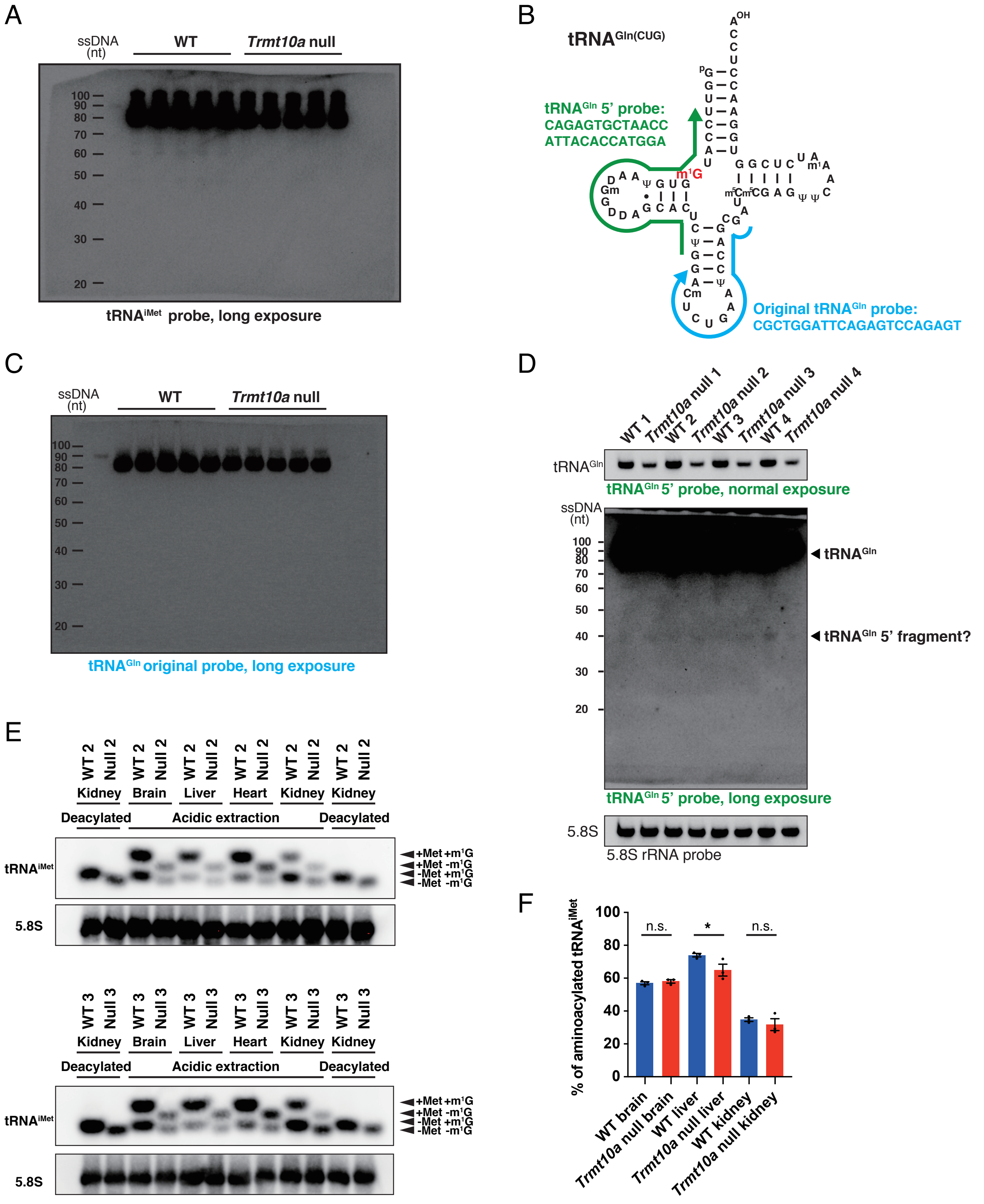

Supplementary Figure 2. Northern blot analysis of *Trmt10a* null and WT mouse brains.

(A) Long exposure image of a whole membrane northern blot conducted to detect tRNA^{iMet} (related to Figure 2B). The positions of the single-stranded DNA (ssDNA) size markers are shown on the left. Note that ssDNA runs slightly faster than RNA of the same length. **(B)** Northern blot probes used to detect tRNA^{Gln}. The northern blot probe for tRNA^{Gln} used in Figure 2B–G is designed to target the region around the anticodon region, and is depicted as the blue "Original tRNA^{Gln} probe". An additional tRNA^{Gln} northern probe (used in D below) was designed to target the 5' half of tRNA^{Gln}, and is depicted as the green "tRNA^{Gln} 5' probe". **(C)** Long exposure image of the tRNA^{Gln} northern blot (related to Figure 2B). **(D)** Northern blot analysis of tRNA^{Gln} from 15-week-old (adult) male mouse brains, conducted using the tRNA^{Gln} 5' region probe depicted in B. Normal exposure (above) and long exposure (below) images are shown. **(E)** Aminoacyl-tRNA northern blot of tRNA^{iMet} and 5.8S rRNA (loading control). The results from $n = 2$ WT and $n = 2$ *Trmt10a* null 15–16-week-old male mice are shown. **(F)** Quantification of aminoacylated tRNA versus non-aminoacylated tRNA from $n = 3$ WT and $n = 3$ *Trmt10a* null mice (in E and Figure 2I) are shown. n.s., not significant or $^*P < 0.05$ (2-way ANOVA followed by Sidak's multiple comparison test).

Supplementary Figure 3. Generation of a human *TRMT10A* KO cell line, and measurement of nascent protein synthesis.

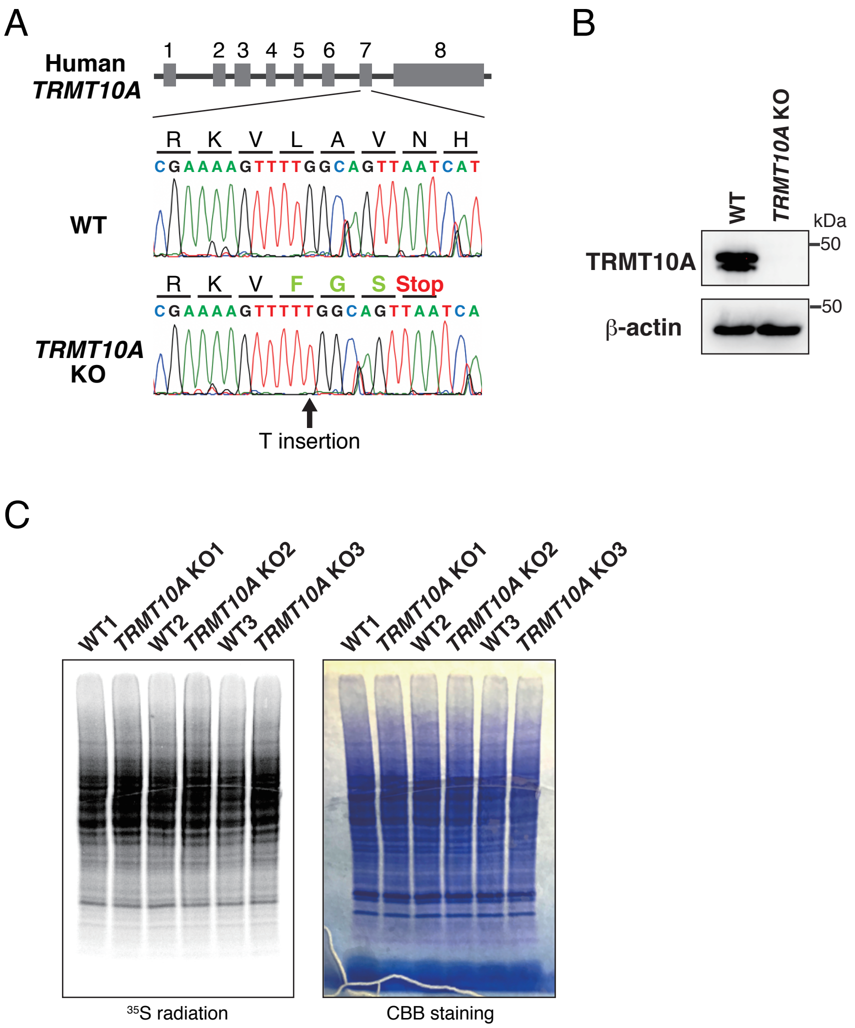

Supplementary Figure 3. Generation of a human *TRMT10A* KO cell line, and measurement of nascent protein synthesis.

(A) Sanger sequencing of *TRMT10A* alleles from WT and *TRMT10A* KO HEK293FT cells. In *TRMT10A* KO cells, both alleles contained a T insertion, resulting in a frameshift and generation of a premature termination codon. **(B)** Western blot analysis of WT and *TRMT10A* KO HEK293FT cellular proteins, conducted to confirm loss of the TRMT10A protein from KO cells. **(C)** Synthesis of nascent cellular proteins was examined using ^{35}S-methionine pulse-labeling. Radiative images of the electrophoresed gel are shown on the left, and CBB-staining of the same gel is shown on the right (loading control). A quantified graph is shown in Figure 4J.

Supplementary Figure 4. Ribosome profiling analysis of *Trmt10a* null and WT mouse brains.

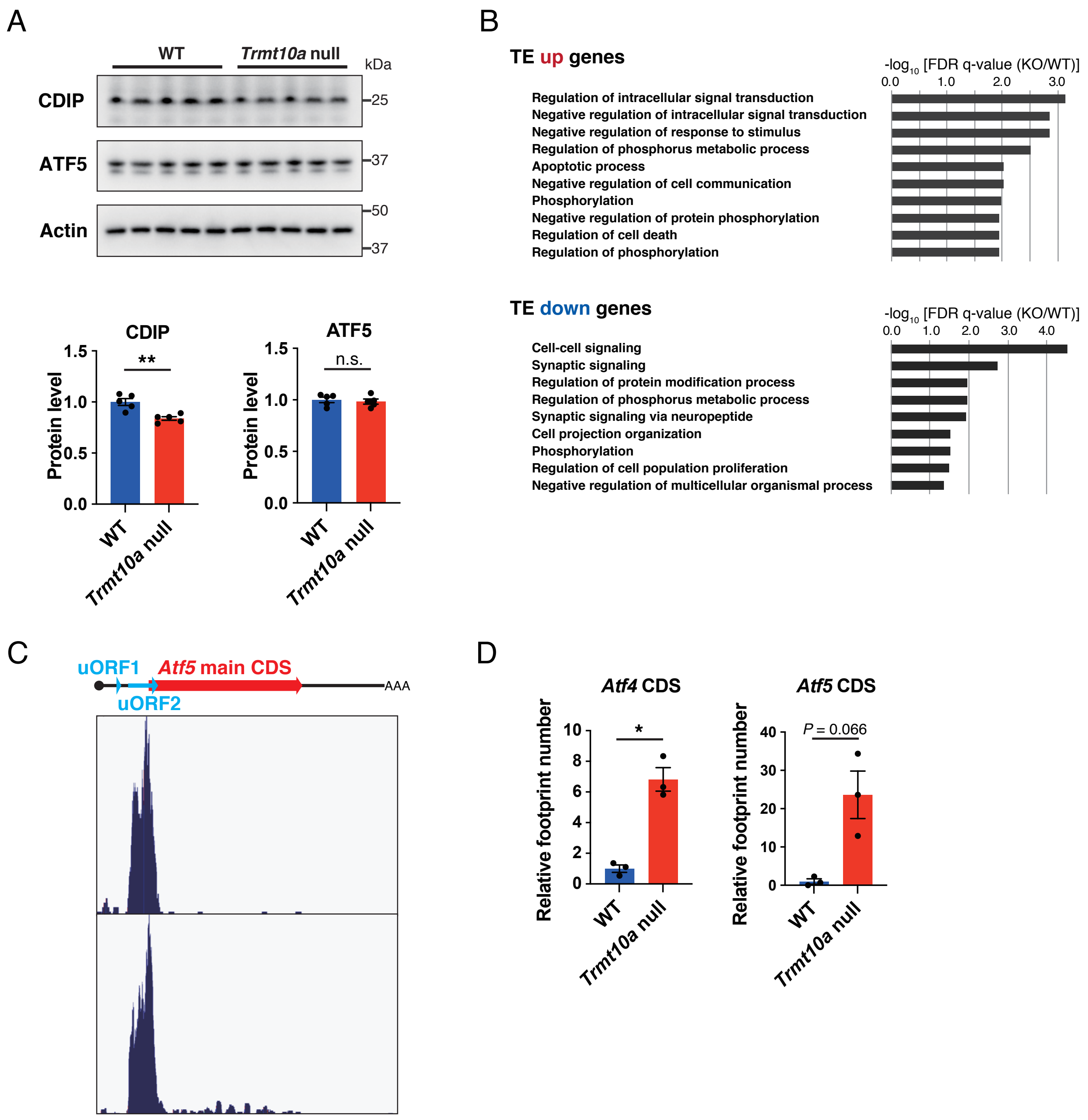

Supplementary Figure 4. Ribosome profiling analysis of *Trmt10a* null and WT mouse brains.

(A) Western blot analysis of CDIP and ATF5 proteins in the brains of $n = 5$ WT and $n = 5$ *Trmt10a* null 15–17-week-old male mice. *Cdip* = TE down gene. Protein levels were normalized to actin, and are depicted at the bottom (data are presented as the mean ± s.e.m.) $^{**}P < 0.01$ or n.s., not significant (Welch’ s *t*-test). **(B)** Gene ontology biological processes analysis of the TE up genes (the red dots in Figure 4C) and TE down genes (the blue dots in Figure 4C). **(C)** Ribosome footprint on *Atf5* mRNAs. Reads derived from ribosome profiling of $n = 3$ WT and $n = 3$ *Trmt10a* null brains (see Figure 4) were added for visibility. **(D)** Quantification of the ribosome footprints on *Atf4* and *Atf5* mRNA CDS, excluding the region that overlaps the uORF. The number of footprints on *Atf4* or *Atf5* mRNA reads from ribosome profiling of $n = 3$ WT and $n = 3$ *Trmt10a* null brains (see Figure 4) was normalized to number of ribosome footprints on *actin* mRNA (data are presented as the mean ± s.e.m. $^{*}P < 0.05$ (Welch’ s *t*-test).

Supplementary Table 1. m^1G9 levels in human tRNAs, as reported in Clark *et al.* RNA (2016)

tRNA (anticodon)	N9 base	m^1G9	N12 base	N23 base
Ala (AGC)	A	n.a.	U	A
Ala (CGC)	A	n.a.	U	A
Ala (TGC)	A	n.a.	U	A
Cys (GCA)	A	n.a.	U	A
Asp (GUC)	A	n.a.	A	U
Glu (CUC)	**G**	**+**	C	G
Glu (UUC)	**G**	**++**	C	G
Phe (GAA)	A	n.a.	U	A
Gly (CCC)	**G**	**-**	U	A
Gly (GCC)	**G**	**-**	U	A
Gly (UCC)	**G**	**-**	A	C
His (GUG)	C	n.a.	A	U
Ile (AAU)	A	n.a.	U	A
Ile (UAU)	**G**	**+++**	G	C
Lys (CUU)	A	n.a.	U	A
Lys (UUU)	A	n.a.	U	A
Leu (AAG)	**G**	**-**	C	G
Leu (CAG)	**G**	**-**	C	G
Leu (UAG)	**G**	**-**	C	G
Leu (CAA)	**G**	**-**	C	G
Leu (UAA)	**G**	**-**	C	G
Met (CAU), initiator	**G**	**+++**	G	C
Met (CAU), elongator	A	n.a.	G	C
Asn (GUU)	**G**	**+++**	G	C
Pro (AGG)	**G**	**+++**	C	G
Pro (CGG)	**G**	**+++**	C	G
Pro (UGG)	**G**	**+++**	C	G
Gln (CUG)	**G**	**+++**	G	C
Gln (UUG)	**G**	**+++**	G	C
Arg (ACG)	**G**	**++**	G	C
Arg (CCG)	**G**	**+++**	C	G
Arg (UCG)	**G**	**+++**	C	G
Arg (CCU)	**G**	**+++**	C	G
Arg (UCU)	**G**	**+++**	G	C
Sec (UCA)	**G**	**-**	C	G
Ser (AGA)	**G**	**-**	C	G
Ser (CGA)	**G**	**-**	C	G
Ser (UGA)	**G**	**-**	C	G
Ser (GCU)	**G**	**-**	C	G
Thr (AGU)	**G**	**++**	U	A
Thr (CGU)	**G**	**+++**	U	A
Thr (UGU)	**G**	**+++**	U	A
Val (AAC)	A	n.a.	G	C
Val (CAC)	A	n.a.	G	C
Val (UAC)	A	n.a.	G	C
Trp (CCA)	**G**	**++**	G	C
Tyr (GUA)	A	n.a.	U	A

+++, ++, +, - indicates the m^1G level in each tRNA species, as previously reported (Clark et al. RNA. 2016). n.a., m^1G level is not available because N9 is not a G. Information about the N12 and N23 bases (Chan et al. NAR. 2016) is provided for reference because N9, N12, and N23 often form base triplets.

Supplementary Table 2. Oligo DNAs used in this study

Name	Sequence
Mouse *Trmt10a* genotyping PCR forward	TCCTTCGAGGACAGTCAATG
Mouse *Trmt10a* genotyping PCR reverse	GCATGCTCTGTGTACGCACT
Mouse tRNA^{iMet} purification	ATCGACCTCTGGGTTATGGGCCCAGCACG 3'Bio
Mouse/human tRNA^{iMet} northern probe	ATCGACCTCTGGGTTATGGGCC
Mouse/human tRNA^{Gln} northern probe	CGCTGGATTCAGAGTCCAGAGT
Mouse tRNA^{Gln} 5' northern probe	CAGAGTGCTAACCATTACACCATGGA
Mouse/human tRNA^{Phe} northern probe	CGAAACCCGGGATCGAACCAGGGACCTTTA
Mouse/human tRNA$^{Arg(UCU)}$ northern probe	CCTTTGAATTAGAAGTCCAATGCGCT
Mouse/human 5.8S rRNA northern probe	GCAAGTGCGTTCGAAGTGTCGATGATCAAT
Ribo-seq/tRNA-seq 3' adaptor	5'rApp/CTGTAGGCACCATCAAT/ddC3'
Ribo-seq/tRNA-seq reverse transcription	5'Phos/AGATCGGAAGAGCGTCGTGTAGGGAAAGAGTGT/iSp18/CAA<br>GCAGAAGACGGCATACGAGATATTGATGGTGCCTACAG
Library PCR/qPCR forward	CAAGCAGAAGACGGCATACGA
Library PCR reverse 1<br>(for tRNA-seq WT1; ribo-seq WT1)	AATGATACGGCGACCACCGAGATCTACACGATCGGAAGAGCACAC<br>GTCTGAACTCCAGTCACACAGTGACACTCTTTCCCTACACGACGC
Library PCR reverse 2<br>(for tRNA-seq WT2; ribo-seq WT2)	AATGATACGGCGACCACCGAGATCTACACGATCGGAAGAGCACAC<br>GTCTGAACTCCAGTCACGCCAATACACTCTTTCCCTACACGACGC
Library PCR reverse 3<br>(for tRNA-seq WT3; ribo-seq WT3)	AATGATACGGCGACCACCGAGATCTACACGATCGGAAGAGCACAC<br>GTCTGAACTCCAGTCACCTTGTAACACTCTTTCCCTACACGACGC
Library PCR reverse 4<br>(for tRNA-seq WT4; ribo-seq Null 1)	AATGATACGGCGACCACCGAGATCTACACGATCGGAAGAGCACAC<br>GTCTGAACTCCAGTCACGTGAAACCACTCTTTCCCTACACGACGC
Library PCR reverse 5<br>(for tRNA-seq Null 1; ribo-seq Null 2)	AATGATACGGCGACCACCGAGATCTACACGATCGGAAGAGCACAC<br>GTCTGAACTCCAGTCACCGATGTACACTCTTTCCCTACACGACGC
Library PCR reverse 6<br>(for tRNA-seq Null 2; ribo-seq Null 3)	AATGATACGGCGACCACCGAGATCTACACGATCGGAAGAGCACAC<br>GTCTGAACTCCAGTCACTGACCAACACTCTTTCCCTACACGACGC
Library PCR reverse 7<br>(for tRNA-seq Null 3)	AATGATACGGCGACCACCGAGATCTACACGATCGGAAGAGCACAC<br>GTCTGAACTCCAGTCACCAGATCACACTCTTTCCCTACACGACGC
Library PCR reverse 8<br>(for tRNA-seq Null 4)	AATGATACGGCGACCACCGAGATCTACACGATCGGAAGAGCACAC<br>GTCTGAACTCCAGTCACCCGTCCCCACTCTTTCCCTACACGACGC
Library qPCR reverse	AATGATACGGCGACCACCGA
Human *TRMT10A* sgRNA top	CACCGGATGAATAGTCGAAAAGTTT
Human *TRMT10A* sgRNA bottom	AAACAAACTTTTCGACTATTCATCC
Human around sg*TRMT10A* PCR forward	TCAACACCAAAAAGTTAACACCA
Human around sg*TRMT10*A PCR reverse	CCAAATGAGCAGTAAAACACACA

Supplementary Table 3. Antibodies used for western blotting

Antibody	Animal, Producer, Catalog number, Dilution
eIF2 alpha	Rabbit, Cell Signaling, 5324T, 1:1000
Phosphorylated eIF2 alpha	Rabbit, Cell Signaling, 3597S, 1:1000
ATF4	Rabbit, Cell Signaling, D4B8, 1:1000
ATF5	Rabbit, Abcam, ab184923, 1:1000
CDIP	Rabbit, Cell Signaling, 13824, 1:500
Beta actin	Mouse, MBL, M177-3, 1:5000
Human TRMT10A	Rabbit, Proteintech, 17294-1AP, 1:1000
Anti-mouse, HRP-conjugated	Goat, Dako, P0447, 1:10,000
Anti-rabbit, HRP-conjugated	Goat, Invitrogen, 31560, 1:4000

Supplementary Table 4. Abbreviations for the modified nucleosides in Figure 1H and I

Modified nucleoside name	Abbreviation
N^1-methyladenosine	m^1A
N^6-threonylcarbamoyladenosine	t^6A
2-methylthio-N^6-threonylcarbamoyladenosine	ms^2t^6A
N^6-isopentenyladenosine	i^6A
2-methylthio-N^6-isopentenyladenosine	ms^2i^6A
Inosine	I
1-methylinosine	m^1I
5-carbamoylmethyluridine	ncm^5U
5-methoxycarbonylmethyl-2-thiouridine	mcm^5s^2U
5-methoxycarbonylmethyl-2'-*O*-methyluridine	mcm^5Um
3-(3-amino-3-carboxypropyl)uridine	acp^3U
5-taurinomethyluridine	τm^5U
5-taurinomethyl-2-thiouridine	τm^5s^2U
2'-*O*-methyluridine	Um
Pseudouridine	Ψ
Dihydrouridine	D
N^1-methylguanosine	m^1G
N^2-methylguanosine	m^2G
7-methylguanosine	m^7G
N^2,N^2-dimethylguanosine	m^2_2G
2'-*O*-methylguanosine	Gm
3-methylcytidine	m^3C
5-methylcytidine	m^5C
N^4-acetylcytidine	ac^4C
5-formylcytidine	f^5C
2'-*O*-methylcytidine	Cm
